# Mental health of couples affects fertility, modified by socioeconomic status: a couple-based analysis

**DOI:** 10.1093/hropen/hoaf071

**Published:** 2025-11-14

**Authors:** Bingjie Wu, Xiaoyue Cheng, Ruimin Zheng, Hua Yang, Mi Xiang, Wei Qiu, Shuai Yang, Kadila Abulaiti, Jiahao Wu, Wenjuan Wang, Fan Jiang, Jinsong Zhang, Jun Zhang

**Affiliations:** Ministry of Education-Shanghai Key Laboratory of Children’s Environmental Health, Xinhua Hospital, Shanghai Jiao Tong University School of Medicine, Shanghai, China; School of Public Health, Shanghai Jiao Tong University School of Medicine, Shanghai, China; Center for Reproductive Medicine, Renji Hospital, Shanghai Jiao Tong University School of Medicine, Shanghai, China; National Center for Women and Children’s Health, National Health Commission, Beijing, China; Hainan Women and Children’s Medical Center, Haikou, China; School of Public Health, Shanghai Jiao Tong University School of Medicine, Shanghai, China; Ministry of Education-Shanghai Key Laboratory of Children’s Environmental Health, Xinhua Hospital, Shanghai Jiao Tong University School of Medicine, Shanghai, China; School of Public Health, Shanghai Jiao Tong University School of Medicine, Shanghai, China; Shanghai Key Laboratory of Embryo-Originated Diseases, International Peace Maternity and Child Health Hospital, Shanghai Jiao Tong University School of Medicine, Shanghai, China; Ministry of Education-Shanghai Key Laboratory of Children’s Environmental Health, Xinhua Hospital, Shanghai Jiao Tong University School of Medicine, Shanghai, China; School of Public Health, Shanghai Jiao Tong University School of Medicine, Shanghai, China; Shanghai Key Laboratory of Embryo-Originated Diseases, International Peace Maternity and Child Health Hospital, Shanghai Jiao Tong University School of Medicine, Shanghai, China; Department of Reproductive Medicine, Xinhua Hospital, Shanghai Jiao Tong University School of Medicine, Shanghai, China; Ministry of Education-Shanghai Key Laboratory of Children’s Environmental Health, Xinhua Hospital, Shanghai Jiao Tong University School of Medicine, Shanghai, China; Shanghai Children’s Medical Center, Shanghai Jiao Tong University School of Medicine, Shanghai, China; Ministry of Education-Shanghai Key Laboratory of Children’s Environmental Health, Xinhua Hospital, Shanghai Jiao Tong University School of Medicine, Shanghai, China; Department of Clinical Psychology, Xinhua Hospital, Shanghai Jiao Tong University School of Medicine, Shanghai, China; Ministry of Education-Shanghai Key Laboratory of Children’s Environmental Health, Xinhua Hospital, Shanghai Jiao Tong University School of Medicine, Shanghai, China; School of Public Health, Shanghai Jiao Tong University School of Medicine, Shanghai, China; Shanghai Key Laboratory of Embryo-Originated Diseases, International Peace Maternity and Child Health Hospital, Shanghai Jiao Tong University School of Medicine, Shanghai, China

**Keywords:** mental health, couple, fertility, preconception, socioeconomic status

## Abstract

**STUDY QUESTION:**

Does preconception mental health status in either partner affect fertility and infertility, and is this association modified by socioeconomic status (SES)?

**SUMMARY ANSWER:**

Preconception mental health problems in both partners are associated with lower couple fertility, with the synergistic impact being most pronounced among couples with low SES status.

**WHAT IS KNOWN ALREADY:**

Mental health problems are rising among young adults, and fertility rates are declining. Women’s preconception mental health has been linked to lower fertility, but few studies have examined the combined impact of both partners’ mental health. The modifying role of SES in these associations is also poorly understood.

**STUDY DESIGN, SIZE, DURATION:**

This couple-based prospective cohort study included 966 preconception couples who sought preconception care and were followed for 12 months in the Shanghai Birth Cohort between 2013 and 2015.

**PARTICIPANTS/MATERIALS, SETTING, METHODS:**

The couples’ mental health status was evaluated at enrolment using the Center for Epidemiological Studies-Depression Scale, Zung Self-Rating Anxiety Scale, and Perceived Stress Scale. The outcomes included couple fecundability (measured by the TTP) and infertility (i.e. TTP >12 menstrual cycles). In the partner-specific model, Cox proportional hazards models and logistic regression were used to evaluate the associations between each partner’s depression, anxiety, and stress levels and couples’ fertility. In the couple-based model, cross-classification and quantile g-computation were first applied to identify couples’ joint exposure to specific psychological conditions in relation to fertility. Latent profile analysis (LPA) was then conducted to characterize distinct latent profiles of couples’ overall mental health statuses, followed by Cox proportional hazards models and logistic regression to examine the corresponding associations. Key symptoms in the couples’ depression, anxiety, and stress scales were determined by elastic net regression and least absolute shrinkage and selection operator. To assess the potential effect modification of SES on the association between couples’ mental health and fertility, we conducted stratified analyses by male and female partner education levels and household income.

**MAIN RESULTS AND THE ROLE OF CHANCE:**

In the female partner-specific model, a 1 SD increase in depression score was associated with 10% lower fecundability (FOR = 0.90, 95% CI: 0.82, 0.99). Likewise, a 1 SD increase in the stress score was associated with 13% lower fecundability (FOR = 0.87, 95% CI: 0.79, 0.96). Male anxiety was associated with a higher risk of infertility (OR = 1.19, 95% CI: 1.01, 1.42). Stratified analyses showed that depression, anxiety, and stress were significantly associated with lower fecundability among males with an education level <college (FOR = 0.79–0.73) and stress was significantly associated with lower fecundability among females with an education level <college or family income <Chinese Yuan 150 000/year (FOR = 0.77). In the couple-based cross-classification model, couples in which both partners reported stress had a 24% lower fecundability (FOR = 0.76, 95% CI: 0.60, 0.96) compared with couples with no stress symptoms. LPA identified seven distinct patterns of couples’ mental health status. Compared with the ‘Neither partner with symptoms’ group, the ‘Female-high/Male-medium’ group showed 56% lower fecundability, followed by 23% lower fecundability in the ‘Female-medium/Male-medium’ group. The greatest attributable impact of the couples’ mental health composite score was observed among couples in which both partners had <college education, accounting for 19.0% of infertility cases.

**LIMITATIONS, REASONS FOR CAUTION:**

Potential reporting and recall biases, residual confounding from unmeasured factors such as antidepressant or other medication use, and prior history of depression, anxiety, and stress were not controlled for. Additionally, mental health was assessed only at baseline, limiting our ability to examine the effects of longitudinal changes in mental health status on fertility outcomes.

**WIDER IMPLICATIONS OF THE FINDINGS:**

This study highlights the importance of addressing the mental health of both partners before conception to improve fertility, particularly among couples with lower SES. Integrating mental health care into preconception services may help lower infertility and related disparities.

**STUDY FUNDING/COMPETING INTEREST(S):**

This study was partly supported by the National Key R&D Program of China (2023YFC2705501 and 2023YFC3905203) and the National Natural Science Foundation of China (41991314). All authors declare no conflict of interest.

**TRIAL REGISTRATION NUMBER:**

N/A.

WHAT DOES THIS MEAN FOR PATIENTS? This study looked at whether the emotional well-being of both partners before pregnancy affects the time taken to become pregnant and the likelihood of infertility, and whether these links differ by education or income level.

We followed 966 couples for 1 year and measured signs of depression, anxiety, and stress in both partners. We found that when either partner had emotional difficulties, the couple was less likely to conceive. When both partners had problems, the chance of becoming pregnant was even lower. These effects were stronger among couples with lower education or incomes.

Our results suggest that mental health care before pregnancy should include both partners, not just females. Providing emotional support to couples planning a pregnancy may help them conceive more easily and reduce differences between families with different income and education levels.

## Introduction

Mental health issues have become increasingly prevalent among young people worldwide over the past two to three decades (Ferrari *et al.*, 2024; Tian *et al.*, 2025). Globally, depressive and anxiety disorders are the most common mental health conditions in individuals aged 25–49 years, ranking as the third and sixth leading causes of disability-adjusted life years (DALYs) in 2019, respectively (GBD 2019 Mental Disorders Collaborators, 2022). At the same time, fertility rates have been declining in most countries, with more than half now falling below replacement levels (Bhattacharjee *et al.*, 2024). This trend signals impending population shrinkage and rapid aging in these regions. Given these challenges, both mental health and fertility have emerged as urgent public health priorities.

Numerous epidemiological studies have examined the association between psychological factors (stress, anxiety, and depression) and fertility among women attempting natural conception; however, the findings remain inconsistent (Braverman *et al.*, 2024). These conditions can disrupt the neuroendocrine system, thereby reducing fertility by decreasing libido and sexual frequency (Indirli *et al.*, 2023; Liao *et al.*, 2024; Mbiydzenyuy and Qulu, 2024). They may also influence sleep, diet, and physical activity, indirectly contributing to subfecundity (Hakimi and Cameron, 2017; Pandi-Perumal *et al.*, 2020; Philippot *et al.*, 2022). Therefore, the inconsistencies in the literature may be attributable to the fact that most previous research has focused primarily on women’s psychological health (Hjollund *et al.*, 1999; Lynch *et al.*, 2012, 2014; Nillni *et al.*, 2016) or investigated a single psychological condition in isolation (Hjollund *et al.*, 1999; Lynch *et al.*, 2014; Evans-Hoeker *et al.*, 2018; Wesselink *et al.*, 2018; Golovina *et al.*, 2023; Liao *et al.*, 2024). At the same time, few studies have included assessments of both partners’ mental health in relation to fertility outcomes. These gaps highlight the need to consider both partners simultaneously when examining the relationship between mental health and fertility.

Couples typically share common living environments, lifestyles, and psychological conditions, and previous research has demonstrated substantial interdependence between partners regarding health behaviours and depressive symptoms (Yang *et al.*, 2023). The mental health of one partner can significantly influence that of the other (Tower and Kasl, 1995). As fertility outcomes inherently involve both partners, investigating the combined effects of male and female psychological health may provide deeper insight into the relationship between mental health and couples’ fertility.

Socioeconomic status (SES) is an indicator of an individual’s economic and social position, representing a broad index of social and environmental conditions that influence many health outcomes and may also affect infertility. SES is commonly defined based on income, education level, or occupation, all of which are relevant components of the SES construct (Jørgensen *et al.*, 2023). SES influences both mental and reproductive health (Jørgensen *et al.*, 2023; Balsara *et al.*, 2025). Stratified analysis by SES allows us to examine its potential modifying role in the association between couples’ mental health and infertility, which may help identify subgroups at higher risk and enable more targeted interventions. To our knowledge, no previous study has explored this potential effect modification.

We investigated the association between couples’ mental health status and their fertility in a large, prospective, couple-based cohort study. Our objectives were: (i) to examine the individual associations of depression, anxiety, and stress experienced by each partner with couple fertility; (ii) to evaluate the combined effects of couples’ depression, anxiety, and stress on fertility; (iii) to develop a composite score integrating key symptoms from the depression, anxiety, and stress scales of both partners, thereby facilitating early identification and targeted intervention; and (iv) to explore the effect modification of SES on the relationship between mental health and couple fertility.

## Materials and methods

### Data source and participants

Between 2013 and 2015, the preconception subcohort of the Shanghai Birth Cohort recruited couples planning pregnancy who visited two preconception care clinics in Shanghai, China. Eligible couples met the following inclusion criteria: (i) aged 20 years or older; (ii) intending to reside continuously in Shanghai for at least 2 years; and (iii) planning spontaneous conception. Exclusion criteria included couples who had (i) been unsuccessfully attempting spontaneous conception for more than 1 year, or (ii) previously sought medical intervention for infertility (Zhang *et al.*, 2019). A total of 1179 preconception couples were initially enrolled. All participants were interviewed to gather information on demographic characteristics, health behaviours, residential environments, and reproductive and medical histories, and participants provided biospecimens. Couples were followed up by telephone every 2 months to obtain the self-reported information, including the pregnancy outcome and contraception time. The pregnancy outcome was collected by a single-choice question (Have you been pregnant since last follow-up? 0 = no; 1 = yes and still pregnant; 2 = yes but miscarried). Information on contraception was collected by a multiple-choice question (Do you currently use any contraceptive methods? 0 = none; 1 = safe-period; 2 = hormonal contraceptive; 3 = short-acting contraceptive; 4 = long-acting contraceptive; 5 = injectable contraceptive; 6 = emergency contraceptives; 7 = condom; 8 = spermicide; 9 = intra-uterine device; 10 = coitus interruptus; 11 = others). The days of contraception were calculated by multiplying the contraceptive times in months by 30 days. The follow-up was no more than 12 months or until pregnancy was achieved. After exclusions, this study included 966 couples who lived together and who had completed mental health assessments (Supplementary Fig. S1). Our study was approved by the Ethics Committee of Xinhua Hospital, Shanghai Jiao Tong University School of Medicine (Approval Number: XHEC-C-2013-001). Signed informed consent was obtained from all participants prior to their enrolment.

### Exposure

Mental health was assessed using the Center for Epidemiological Studies-Depression Scale (CES-D) for depression (Radloff, 1977), the Zung self-rating Anxiety Scale (SAS) for anxiety (Zung, 1971), and the Perceived Stress Scale (PSS) for stress (Cohen *et al.*, 1983). The Chinese versions of these three standard instruments had been calibrated with good validity (Lau *et al.*, 2010; Wang *et al.*, 2011). CES-D was further categorized into three classes: ‘no depression symptoms’ (<16), ‘depressive tendency’ (16–19), and ‘in depression status’ (≥20). The SAS score was used to classify participants into ‘without anxiety symptoms’ (<50), ‘in mild anxiety status’ (50–59), ‘in moderate anxiety status’ (60–69), and ‘in serious anxiety’ (≥70). Total PSS scores of 15 or higher were considered elevated stress. In this study, couples were cross-classified according to the cutoff values of CES-D ≥ 20, SAS ≥50 and PSS ≥15 into the following groups: none (neither partner’s score in the given dimension exceeded the cutoff), male only (the male partner’s score in the given dimension exceeded the cutoff), female only (the female partner’s score in the given dimension exceeded the cutoff), and both (both partners’ scores exceeded the cutoff in the given dimension).

### Outcomes

Outcomes included couple fecundability and infertility. Couple fecundability was assessed by TTP. For the pregnant and non-pregnant couples, TTP was calculated by Equations (1) and (2), respectively.


(1) $$\text{TTP}=\frac{D_{3}-D_{1}-D_{2}}{A}$$

(2) $$\text{TTP}=\frac{D_{4}-D_{1}-D_{2}}{A}$$

Where *D*1 is the date of enrolment, *D*2 is the days of contraception, *D*3 is the date of the last menstrual period, *D*4 is the date of the last follow-up, and *A* is the average menstrual cycle length collected at enrolment. For females with irregular menstrual cycles, the average menstrual cycle length was calculated as the mean of the longest and shortest menstrual cycles. We added one menstrual cycle to the TTP of those who were pregnant during follow-up (Hong *et al.*, 2019; Luo *et al.*, 2022). Couple infertility is defined as a TTP >12 menstrual cycles. Couples with TTP <12 menstrual cycles and not pregnant were excluded from the infertility analysis. The days of contraception were calculated by multiplying the contraceptive duration in months by 30 days. In this study, we use ‘fecundability’ to represent the probability of conception related to TTP, for which we fitted a Cox proportional hazards model to estimate the association between couples’ mental health and time to conception. ‘Infertility’ denotes the inability to conceive, analysed as a binary outcome (defined as TTP >12 menstrual cycles) using logistic regression. We use ‘fertility’ as a shorthand term encompassing both measured outcomes (Zegers-Hochschild *et al.*, 2017).

### Covariates

At enrolment, information was collected using a structured questionnaire administered by trained interviewers, which included demographic characteristics, reproductive history, lifestyle factors, medical history, and disease history. Potential confounders were selected for adjustment based on a directed acyclic graph. Previous studies included ages (years), BMI (kg/m^2^), family income [<Chinese Yuan (CNY) 150 000/year, CNY 150 000–CNY 300 000/year, >CNY 300 000/year], education level (<college, college, >college), smoking (yes, no), drinking (yes, no), age of menarche (<13 years, ≥13 years), pregnancy history (had, not had), live birth history (had, not had), miscarriage history (had, not had), induced abortion history (had, not had), and parity (nulliparous, parous) (Supplementary Fig. S2). In the couple-based models, we adjusted for the female’s age and the age difference between partners to account for the high correlation between male and female age.

### Statistical analysis

Demographic characteristics were described as median [interquartile range] and n (%) for continuous and categorical covariates, respectively. First, we performed individual-specific analyses to assess the associations between each partner’s depression, anxiety, and stress and the couples’ fertility, and further explored potential effect modification by SES. Second, considering the potential impact of shared depression, anxiety, and stress in couples on their fertility, we performed couple-based analyses to assess the effects of co-exposure to depression, anxiety, and stress in couples on fertility (Luo *et al.*, 2022; Ao *et al.*, 2023), while also examining whether these associations were modified by SES.

Fecundability odds ratio (FOR) and 95% CI between mental health scores (including specific symptoms) and TTP were estimated by a discrete-time Cox proportional hazards model. FOR >1.0 represents a higher fecundability, whereas FOR <1.0 refers to lower fecundability. For infertility, a logistic regression model was used to estimate the odds ratio (OR) and its 95% CI. OR represents the impact of each SD increase in mental health scores on infertility risk, with OR >1.0 indicating a higher risk of infertility, while OR <1.0 indicates a decreased risk. To model the non-linear relationships between mental health scores and couple fertility, a multivariable restricted cubic spline (RCS) method was applied. A Wald test was used to assess the significance of the RCS model. The joint effect of couples’ depression, anxiety, and stress continuous scores mixture on couple fertility was assessed using the quartile-based g-computation (q-gcomp) method. Distinct patterns of couples’ depression, anxiety, and stress continuous *Z*-scores were assessed using the latent profile analysis (LPA). We chose the best-fitting LPA model for couple mental health profile according to the following criteria: (i) model entropy close to 1.0; (ii) lowest Bayesian information criterion; (iii) lowest Akaike information criterion; (iv) bootstrap likelihood ratio test *P *< 0.05 compared to n−1 model; (v) acceptable proportion of the population (≥3.0%); and (vi) prior knowledge of research. Cox proportional hazards models and logistic regression were performed to explore the relationship between distinct couples’ mental health indicators’ patterns and fertility. We also constructed elastic net regression (ENR) to select responses to couples’ mental health subdomain questions related to fertility. Of the 100 variables, 14 of the most importance were identified by the ENR and calculated as a couple’s mental health composite score. Further RCS analysis was performed to determine the optimal cut-off value for the couples’ mental health composite score in fertility to be applied in early clinical intervention. Attributable fractions (AFs) were used to measure the burden of exposures on couples’ mental symptoms, including the couples’ mental health composite score, which interprets the decreasing proportion of the population with fertility after exposure intervention. The detailed methods for the estimation of the AF are provided in Supplementary Materials and Methods. Male and female partner education level (<college or ≥college), joint education level (both <college, one of the couples <college, or both ≥college), and family income (<CNY 150 000/year or ≥CNY 150 000/year) were used as SES indicators to explore the potential effect modification by SES on the association between couples’ mental health and fertility.

Several sensitivity analyses were performed to assess the robustness of our results. First, missing data were imputed using multiple imputation by chained equations (MICE). Second, to assess the effects of unreported attempting time before enrolment (left-truncation), we reanalysed the data after censoring TTP at six menstrual cycles (i.e. subfecundability and subfertility). Third, we performed a stratified analysis by menstrual cycle regularity and parity.

RCS (rms package), q-gcomp (qgcomp package), LPA (tidyLPA package), ENR (glmnet package), AF (AF package), and MICE (mice package) were performed in R version 4.4.1 (R Core Team, Vienna, Austria). Hypothesis tests were two-sided, and *P *< 0.05 was considered statistically significant.

## Results

Table 1 presents the demographic characteristics of the pre-conception couples. Females in the non-pregnant group were more likely to be older, have a higher PSS score, and were less likely to have a history of miscarriage, induced abortion, or pregnancy. Males in the non-pregnant group were more likely to be older, and to smoke and drink. Among all participants, 91.5% of the females were nulliparous. During the 12-month follow-up, 569 couples (58.9%) conceived spontaneously.

**Table 1.** Demographic characteristics of the pre-conception couples.

	Total	Pregnant	Non-pregnant	*P*
	(n = 966)	(n = 569)	(n = 397)	
**Female characteristics**				
**Age (years)**	29.6 [27.6; 31.7]	29.4 [27.4; 31.6]	29.9 [27.9; 31.9]	0.018*
**Smoking**				0.544
No	937 (97.0%)	554 (97.4%)	383 (96.5%)	
Yes	29 (3.0%)	15 (2.6%)	14 (3.5%)	
**Drinking**				0.383
No	622 (64.4%)	374 (65.7%)	248 (62.5%)	
Yes	343 (35.5%)	194 (34.1%)	149 (37.5%)	
Missing	1 (0.1%)	1 (0.1%)	0 (0.0%)	
**BMI (kg/m^2^)**	20.5 [19.1; 22.4]	20.6 [19.1; 22.6]	20.4 [19.1; 22.2]	0.205
**Education level**				0.353
<College	191 (19.8%)	109 (19.2%)	82 (20.7%)	
College	581 (60.1%)	337 (59.2%)	244 (61.5%)	
>College	194 (20.1%)	123 (21.6%)	71 (17.9%)	
**Family income (yuan/year)**				0.900
<150 000	217 (22.5%)	129 (22.7%)	88 (22.2%)	
150 000–300 000	515 (53.3%)	303 (53.3%)	212 (53.4%)	
>300 000	184 (19.0%)	110 (19.3%)	74 (18.6%)	
Missing	50 (5.1%)	27 (4.7%)	23 (5.7%)	
**Physical activity levels**				0.898
Low	220 (22.8%)	126 (22.1%)	94 (23.7%)	
Moderate	550 (56.9%)	326 (57.3%)	224 (56.4%)	
High	181 (18.7%)	109 (19.2%)	72 (18.1%)	
Missing	15 (1.5%)	8 (1.4%)	7 (1.7%)	
**Age of menarche (years)**				0.136
<13	200 (20.7%)	128 (22.5%)	72 (18.1%)	
≥13	620 (64.2%)	363 (63.8%)	257 (64.7%)	
Missing	146 (15.1%)	78 (13.7%)	68 (17.1%)	
**History of pregnancy**				0.001*
No	541 (56.0%)	293 (51.5%)	248 (62.5%)	
Yes	425 (44.0%)	276 (48.5%)	149 (37.5%)	
**History of live birth**				0.838
No	907 (93.9%)	533 (93.7%)	374 (94.2%)	
Yes	59 (6.1%)	36 (6.3%)	23 (5.7%)	
**History of miscarriage**				0.011*
No	871 (90.2%)	501 (88.0%)	370 (93.2%)	
Yes	95 (9.8%)	68 (12.0%)	27 (6.8%)	
**History of induced abortion**				0.009*
No	695 (71.9%)	391 (68.7%)	304 (76.6%)	
Yes	271 (28.1%)	178 (31.3%)	93 (23.4%)	
**History of stillbirth**				1.000
No	965 (99.9%)	568 (99.8%)	397 (100%)	
Yes	1 (0.1%)	1 (0.1%)	0 (0.0%)	
**Parity**				0.222
No	884 (91.5%)	515 (90.5%)	369 (92.9%)	
Yes	82 (8.4%)	54 (9.4%)	28 (7.0%)	
**CES-D**	11.0 [6.00; 16.0]	10.0 [6.00; 16.0]	11.0 [7.00; 17.0]	0.051
**SAS**	36.0 [32.0; 41.0]	36.0 [32.0; 41.0]	37.0 [33.0; 41.0]	0.102
**PSS**	15.0 [12.0; 17.0]	15.0 [12.0; 17.0]	15.0 [13.0; 17.0]	0.012*
**CES-D levels**				0.010*
<16	697 (72.2%)	413 (72.6%)	284 (71.5%)	
16∼19	143 (14.8%)	95 (16.7%)	48 (12.1%)	
≥20	126 (13.0%)	61 (10.7%)	65 (16.4%)	
**SAS levels**				0.286
<50	913 (94.5%)	542 (95.3%)	371 (93.5%)	
≥50	53 (5.4%)	27 (4.7%)	26 (6.5%)	
**PSS levels**				0.064
<15	437 (45.2%)	272 (47.8%)	165 (41.6%)	
≥15	529 (54.8%)	297 (52.2%)	232 (58.4%)	
**TTP (menstrual cycles)**	7.0 [4.0; 12.0]	5.0 [3.0; 9.0]	12.0 [8.0; 12.0]	<0.001*
**Male characteristics**				
**Age (years)**	30.8 [28.7; 33.4]	30.6 [28.5; 33.1]	31.1 [28.8; 33.7]	0.039*
**BMI (kg/m^2^)**	23.9 [22.0; 25.7]	23.9 [22.1; 25.7]	23.8 [21.8; 25.8]	0.611
**Smoking**				0.043*
No	652 (67.5%)	395 (69.4%)	257 (64.7%)	
Yes	308 (31.9%)	173 (30.4%)	135 (34.0%)	
Missing	6 (0.6%)	1 (0.1%)	5 (1.2%)	
**Drinking**				0.011*
No	202 (20.9%)	109 (19.2%)	93 (23.4%)	
Yes	757 (78.4%)	459 (80.7%)	298 (75.1%)	
Missing	7 (0.7%)	1 (0.1%)	6 (1.5%)	
**Education level**				0.353
<College	172 (17.8%)	95 (16.7%)	77 (19.4%)	
College	537 (55.6%)	313 (55.0%)	224 (56.4%)	
>College	256 (26.5%)	160 (28.1%)	96 (24.2%)	
Missing	1 (0.1%)	1 (0.1%)	0 (0.0%)	
**CES-D**	10.0 [6.00; 15.0]	10.0 [5.00; 15.0]	10.0 [6.00; 15.0]	0.188
**SAS**	35.0 [30.0; 38.0]	33.0 [30.0; 38.0]	35.0 [31.0; 38.0]	0.073
**PSS**	14.0 [12.0; 17.0]	14.0 [11.0; 17.0]	15.0 [12.0; 17.0]	0.085
**CES-D levels**				0.341
<16	739 (76.5%)	439 (77.2%)	300 (75.6%)	
16∼19	124 (12.8%)	76 (13.4%)	48 (12.1%)	
≥20	103 (10.7%)	54 (9.49%)	49 (12.3%)	
**SAS levels**				0.612
<50	945 (97.8%)	555 (97.5%)	390 (98.2%)	
≥50	21 (2.1%)	14 (2.4%)	7 (1.7%)	
**PSS levels**				0.216
<15	506 (52.4%)	308 (54.1%)	198 (49.9%)	
≥15	460 (47.6%)	261 (45.9%)	199 (50.1%)	

* Values marked with an asterisk denote statistical significance (*P* < 0.05).

Medium [IQR] were compared using the Mann–Whitney U test; n (%) were compared using the chi-squared test. BMI, body mass index; PSQI, Pittsburgh sleep quality index; CES-D, Center for Epidemiological Studies-Depression Scale; SAS, Self-rating Anxiety Scale; PSS, Perceived Stress Scale; TTP, time to pregnancy.

**Figure 1.**
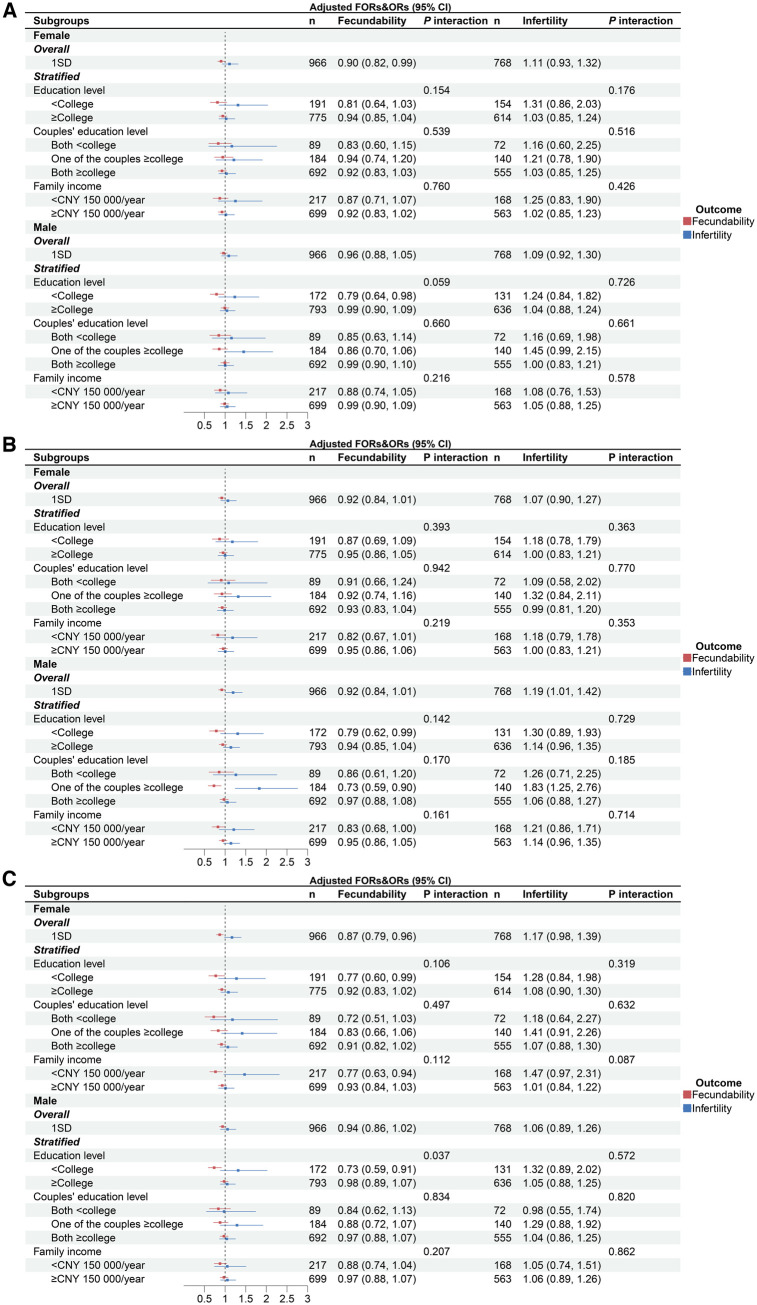
**Associations between each partner’s mental health and fertility by financial and educational subgroups**. (**A**) Depression; (**B**) anxiety; (**C**) stress. FOR, fertility odds ratio; OR, odds ratio; CNY, Chinese Yuan. For females, data were adjusted for their age, body mass index, family income, education level, smoking, drinking, age of menarche, pregnancy history, parity, live birth history, miscarriage history, and induced abortion history. For males, data were adjusted for their age, body mass index, family income, education level, smoking, drinking, and their partner’s pregnancy history, parity, live birth history, miscarriage history, and induced abortion history. Data were adjusted for all the other factors except the stratified factor.

As shown in Fig. 1, in the female partner-specific model, a 1 SD increase in depression score was associated with 10% lower fecundability (FOR = 0.90, 95% CI: 0.82, 0.99). Likewise, a 1 SD increase in stress score was associated with 13% lower fecundability (FOR = 0.87, 95% CI: 0.79, 0.96). Male anxiety was associated with a higher risk of infertility (OR = 1.19, 95% CI: 1.01, 1.42). In the SES-stratified analysis, the male education level modified the associations between mental health conditions (depression, anxiety, and stress) and couple fecundability; specifically, mental health conditions were significantly associated with lower fecundability in males with education level <college (OR = 0.79, 95% CI: 0.64, 0.98 for depression; OR = 0.79, 95% CI: 0.62, 0.99 for anxiety; OR = 0.73, 95% CI: 0.59, 0.91 for stress), but no significant relationship was found between mental health and fecundability in males with education level ≥college. Additionally, among women, education level and family income modified the association between stress and couple fecundability. Stress was significantly associated with lower fecundability among women with an education <college (OR = 0.77, 95% CI: 0.60, 0.99) or family income <CNY 150 000/year (OR = 0.77, 95% CI: 0.63, 0.94). However, no significant association was observed among women with an education level ≥college or a family income ≥CNY 150 000/year. No significant interaction was detected between couples’ education level and mental health in relation to infertility. However, the association between infertility and depression, stress, and anxiety appeared to weaken as couples’ educational attainment increased, from both partners having <college education, to one partner having ≥college education, and to both partners having ≥college education.

The RCS did not find a non-linear association between couples’ mental health and fertility, except for male anxiety (*P* for non-linearity = 0.017) (Supplementary Figs S3 and S4).

**Figure 2.**
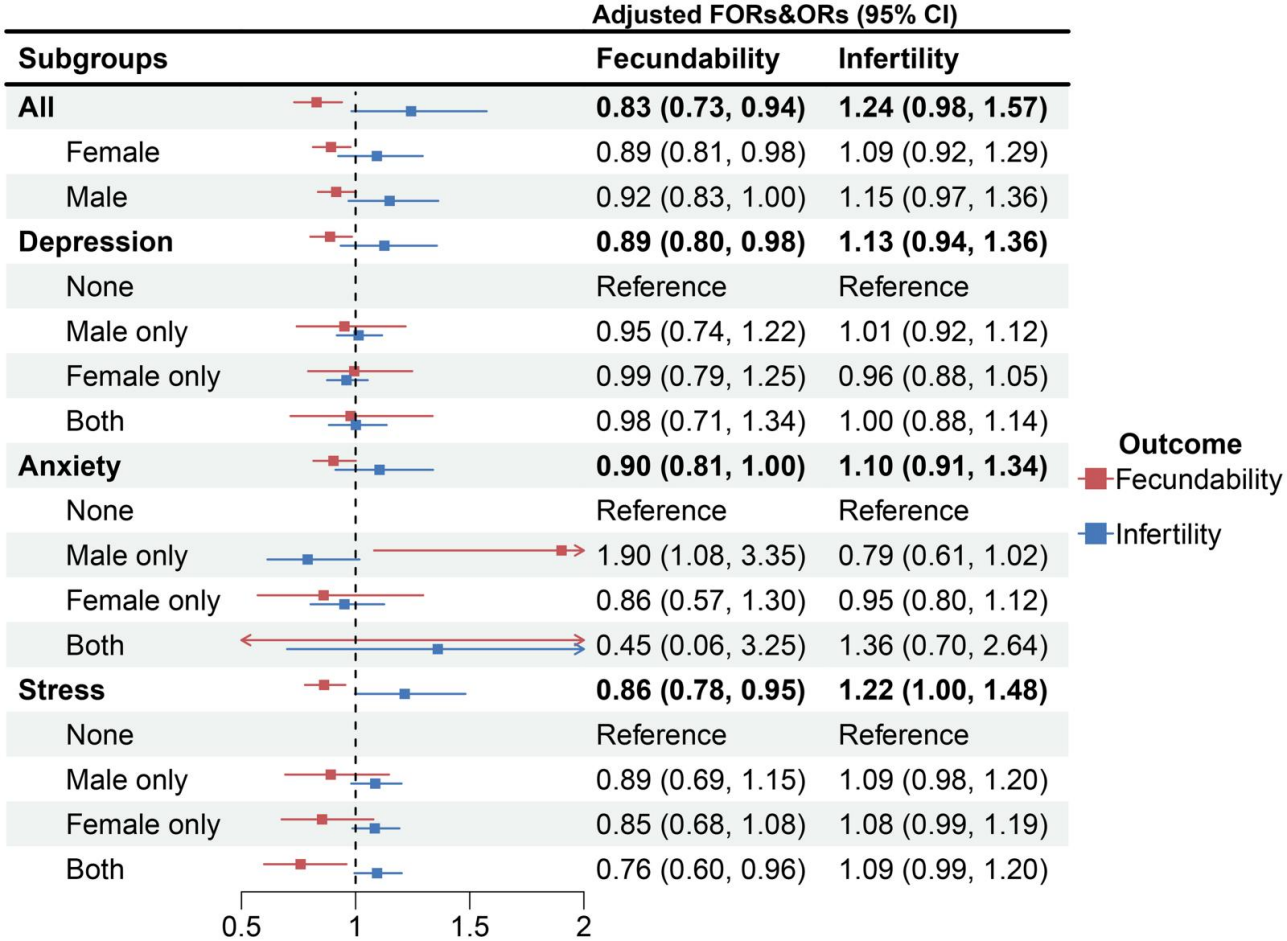
**Associations of couples’ depression, anxiety, and stress with their fertility**. FOR, fertility odds ratio; OR, odds ratio. All, Female, Male, Depression, Anxiety, and Stress represent the associations between mixtures of different couples’ mental health component scores and fertility, calculated using the q-gcomp model. All represents the six components in the mixture (female/male stress, anxiety, and depression); Female represents the three components in the mixture (female stress, anxiety, and depression); Male represents the three components in the mixture (male stress, anxiety, and depression); Depression represents the two components in the mixture (female/male depression); Anxiety represents the two components in the mixture (female/male anxiety); Stress represents the two components in the mixture (female/male stress). None, male only, female only, and both represent subgroups defined by cross-classification according to the cutoff values of the corresponding dimension, analysed using Cox or logistic regression models with None as the reference group. Data were adjusted for female age, difference between female and male ages, body mass index (each partner), family income, smoking (each partner), drinking (each partner), age of menarche, pregnancy history, parity, live birth history, miscarriage history, and induced abortion history.

Figure 2 presents the fully adjusted FOR (95% CIs) and OR (95% CIs) for the associations of couples’ depression, anxiety, and stress mixtures with fecundability and infertility, respectively, as estimated using the q-gcomp model. Significant associations were found between lower fecundability and the overall mental health mixture (including depression, anxiety, and stress for both partners; FOR = 0.83, 95% CI: 0.73, 0.94), the female-specific mixture (FOR = 0.89, 95% CI: 0.81, 0.98), the depression-specific mixture (FOR = 0.89, 95% CI: 0.80, 0.98), and the stress-specific mixture (FOR = 0.86, 95% CI: 0.78, 0.95). Associations between these mental health mixtures and a higher risk of couple infertility were suggestive but did not reach statistical significance.

Furthermore, Fig. 2 also presents the results of the cross-classification based on the cutoff values of the corresponding couples’ mental health dimensions and their associations with fertility. Compared with couples in whom neither partner experienced stress, those in whom either or both partners experienced stress tended to have lower fecundability; however, this lower fecundability was statistically significant only when both partners experienced stress (FOR = 0.76, 95% CI: 0.60, 0.96). We also found that while the association between the mixture of couples’ depression and fertility was significant, the decomposed effect was not, suggesting a potential synergistic effect of couples’ mental health. Surprisingly, compared with couples in whom neither partner had anxiety, couples in whom only the male partner experienced anxiety appeared to have a higher fecundability (FOR = 1.90, 95% CI: 1.08, 3.35).

Tables 2, 3, and 4 and Supplementary Tables S1, S2, and S3 present the SES-stratified results of couples’ mental health mixtures and fertility estimated by the q-gcomp model. Most mixture patterns showed stronger associations among couples with lower SES. This trend was particularly evident for the all mixture, which included all six mental health components from both partners. For fecundability, significant associations were observed in the <college subgroup [female: FOR = 0.64 (95% CI: 0.46, 0.88); male: FOR = 0.64 (95% CI: 0.47, 0.88)]. For infertility, the corresponding associations were also stronger in the <college subgroup [female: OR = 1.18 (95% CI: 1.05, 1.33); male: OR = 1.15 (95% CI: 1.03, 1.28)]. Although the *P* for interaction was not statistically significant, the low-SES subgroups had substantially smaller sample sizes than the high-SES subgroups in our study. The observed differences in effect sizes nonetheless suggest potential effect modification by SES.

**Table 2.** Educational subgroup analyses of the association between couples’ mental health mixture and fecundability (q-gcomp model).

Subgroup	<College	≥College	*P* for interaction
**Stratified by female education**			
All mixture	0.64 (0.46, 0.88)*	0.87 (0.75, 1.01)	0.157
Female mixture	0.74 (0.57, 0.97)*	0.90 (0.80, 1.00)	0.111
Male mixture	0.85 (0.69, 1.04)	0.92 (0.83, 1.02)	0.399
Couple depression mixture	0.75 (0.58, 0.97)*	0.93 (0.83, 1.05)	0.267
Couple anxiety mixture	0.71 (0.54, 0.93)*	0.96 (0.85, 1.08)	0.249
Couple stress mixture	0.71 (0.54, 0.94)*	0.88 (0.78, 0.99)*	0.054
**Stratified by male education**			
All mixture	0.64 (0.47, 0.88)*	0.87 (0.75, 1.01)	0.768
Female mixture	0.78 (0.63, 0.96)*	0.93 (0.84, 1.03)	0.825
Male mixture	0.81 (0.65, 1.01)	0.92 (0.83, 1.03)	0.698
Couple depression mixture	0.75 (0.59, 0.97)*	0.94 (0.83, 1.05)	0.689
Couple anxiety mixture	0.71 (0.55, 0.93)*	0.96 (0.86, 1.08)	0.649
Couple stress mixture	0.71 (0.55, 0.93)*	0.88 (0.78, 0.99)*	0.812

* Values marked with an asterisk denote statistical significance (*P* < 0.05).

Hypothesis test statistics and CIs are based on using the delta estimate variance of a linear combination of random variables.

All mixture represents the six components in the mixture (female/male stress, anxiety, and depression); Female mixture represents the three components in the mixture (female stress, anxiety, and depression); Male mixture represents the three components in the mixture (male stress, anxiety, and depression); Depression mixture represents the two components in the mixture (female/male depression); Anxiety mixture represents the two components in the mixture (female/male anxiety); Stress mixture represents the two components in the mixture (female/male stress). Data were adjusted for female age, difference between female and male ages, body mass index (each partner), family income, smoking (each partner), drinking (each partner), age of menarche, pregnancy history, parity, live birth history, miscarriage history, and induced abortion history.

**Table 3.** Financial subgroup analyses of the association between couples’ mental health mixture and fecundability (q-gcomp model).

Subgroup	<CNY 150 000/year	≥CNY 150 000/year	*P* for interaction
All mixture	0.76 (0.56, 1.01)	0.87 (0.75, 1.00)	0.170
Female mixture	0.78 (0.61, 0.99)*	0.90 (0.80, 1.01)	0.147
Male mixture	0.82 (0.67, 1.01)	0.94 (0.85, 1.05)	0.477
Couple depression mixture	0.89 (0.71, 1.12)	0.91 (0.81, 1.02)	0.933
Couple anxiety mixture	0.81 (0.64, 1.03)	0.95 (0.84, 1.07)	0.055
Couple stress mixture	0.82 (0.64, 1.05)	0.88 (0.78, 0.99)*	0.399

* Values marked with an asterisk denote statistical significance (*P* < 0.05).

Hypothesis test statistics and CIs are based on using the delta estimate variance of a linear combination of random variables.

CNY, Chinese Yuan. All mixture represents the six components in the mixture (female/male stress, anxiety, and depression); Female mixture represents the three components in the mixture (female stress, anxiety, and depression); Male mixture represents the three components in the mixture (male stress, anxiety, and depression); Depression mixture represents the two components in the mixture (female/male depression); Anxiety mixture represents the two components in the mixture (female/male anxiety); Stress mixture represents the two components in the mixture (female/male stress). Data were adjusted for female age, difference between female and male ages, body mass index (each partner), education level (each partner), smoking (each partner), drinking (each partner), age of menarche, pregnancy history, parity, live birth history, miscarriage history, and induced abortion history.

**Table 4.** Couples’ educational subgroup analyses of the association between couples’ mental health mixture and fecundability (q-gcomp model).

Subgroup	Both <College	One of the couples <College	Both ≥College
All mixture	0.57 (0.34, 0.97)*	0.70 (0.52, 0.94)*	0.88 (0.76, 1.01)
Female mixture	0.74 (0.54, 1.02)	0.93 (0.75, 1.15)	0.91 (0.82, 1.01)
Male mixture	0.91 (0.67, 1.24)	0.73 (0.59, 0.91)*	0.95 (0.86, 1.06)
Couple depression mixture	0.76 (0.52, 1.10)	0.83 (0.67, 1.05)	0.92 (0.82, 1.03)
Couple anxiety mixture	0.79 (0.53, 1.16)	0.77 (0.60, 0.97)*	0.94 (0.83, 1.05)
Couple stress mixture	0.77 (0.52, 1.14)	0.81 (0.64, 1.02)	0.90 (0.80, 1.01)

* Values marked with an asterisk denote statistical significance (*P* < 0.05).

Hypothesis test statistics and CIs are based on using the delta estimate variance of a linear combination of random variables.

All mixture represents the six components in the mixture (female/male stress, anxiety, and depression); Female mixture represents the three components in the mixture (female stress, anxiety, and depression); Male mixture represents the three components in the mixture (male stress, anxiety, and depression); Depression mixture represents the two components in the mixture (female/male depression); Anxiety mixture represents the two components in the mixture (female/male anxiety); Stress mixture represents the two components in the mixture (female/male stress). Data were adjusted for female age, difference between female and male ages, body mass index (each partner), family income, smoking (each partner), drinking (each partner), age of menarche, pregnancy history, parity, live birth history, spontaneous abortion history, and induced abortion history.

The couples’ mental health profiles were classified into seven groups (Supplementary Table S4). Supplementary Figure S5 illustrates the seven groups in a line graph. The seven groups comprised 13.3% (Group 1, F low/M medium), 16.1% (Group 2, F medium/M low), 11.5% (Group 3, F medium/M medium), 26.3% (Group 4, Neither partner with symptoms), 22.0% (Group 5, F medium/M medium), 7.2% (Group 6, F low/M high), and 3.5% (Group 7, F high/M medium) of the total sample. Since Group 3 and Group 5 had similar levels of *Z*-scores, we combined them into one group (F medium/M medium).

Supplementary Table S5 shows the association between the mental health profiles of couples and their fecundability and infertility. Compared to couples with neither having symptoms (Group 4), the ‘F high/M medium’ group showed 56% lower fecundability (FOR = 0.44, 95% CI: 0.23, 0.84), followed by the ‘F medium/M medium’ group (FOR = 0.77, 95% CI: 0.62, 0.97), suggesting a dose-response pattern. However, most associations for the F high/M medium and F medium/M medium groups were not statistically significant in the stratified analyses by SES, likely due to reduced sample size. Nevertheless, the point estimates remained lower for fecundability (or higher for infertility) compared with the corresponding estimates in the unstratified analyses (Supplementary Tables S6, S7, S8, and S9).

Supplementary Figure S6 shows the process of selecting the mental health subdomain questions based on couples’ fertility. Fourteen of the 100 most important questions were selected consistently by ENR and were combined to form the new fertility-targeted composite mental health score with a maximum score of 14 points (Supplementary Table S10). Supplementary Figure S7 shows that the optimal cut-off value for the couples’ mental health composite score, determined by RCS analysis, was seven. Supplementary Figure S8 and Supplementary Table S11 further suggest that the mental health composite score above the cut-off value may result in a significant lower couples’ fecundability [−7.9% (−13.7%, −2.1%) to −4.2% (−7.5%, −1.0%)] and higher infertility risk [7.0% (−0.2%, 14.1%)]. Moreover, lower fecundability or higher infertility could not be attributed to the couples’ depression (‘in depression status’, CES-D ≥ 20), anxiety (‘in mild anxiety status’ or ‘in moderate anxiety status’, SAS ≥50), or stress (‘elevated stress’, PSS ≥15) (Supplementary Fig. S9 and Supplementary Table S11). Supplementary Figures S10 and S11 and Supplementary Tables S12 and S13 illustrate that among males and females with education levels below college, lower fecundability [female: −17.1% (−32.6%, −1.6%) to −9.6% (−18.8%, −0.4%); male: −46.3% (−95.5%, 2.8%) to −14.7% (−25.4%, −4.0%)] and higher infertility [female: 10.9% (−1.6%, 23.4%); male: 10.7% (−3.2%, 24.6%)] could be significantly attributed to the couples’ mental health composite score. Furthermore, among couples with family income <CNY 150 000/year, lower fecundability [−14.8% (−27.9%, −1.8%) to −7.8% (−15.1%, −0.5%)] could be significantly attributed to the couples’ mental health composite score. Supplementary Figure S12 and Supplementary Table S14 show that among couples in which both partners had <college education, up to 19.0% (1.4%, 36.6%) of infertility cases and a − 21.4% (−48.1%, 5.3%) to −11.8% (−27.3%, 3.8%) lower fecundability were attributable to poor couples’ mental health composite score. These AFs were higher than those observed in couples where only the male or only the female partner had less than college education.

The associations did not change substantially after data imputation or censoring TTP at six menstrual cycles, and similar results were observed when analyses were restricted to nulliparous couples or females with regular menstrual cycles, suggesting that parity and menstrual cycle regularity did not materially affect our findings (Supplementary Tables S15, S16, S17, S18, S19, S20, S21, S22, S23, S24, S25, and S26; Supplementary Figs S13, S14, and S15).

## Discussion

In this prospective cohort, using complementary approaches (individual-specific models, q-gcomp mixtures, cross-classification, latent profiles, the data-driven composite score, and sensitivity analyses), we observed a consistent pattern: worse couples’ mental health (higher symptom burden) was associated with lower fecundability, whereas associations with infertility were generally weaker. At the individual level, female depression and stress are related to lower fecundability, and male anxiety is linked to higher infertility risk. Couple-based analyses suggested dyadic synergy, with the lowest fecundability when both partners reported stress. Mixture models pointed in the same direction, and latent profiles indicated a dose-response pattern, with profiles reflecting higher combined symptom burdens showing the poorest fecundability. Importantly, SES appeared to modify these associations, which were stronger in lower-education and low-income strata. A concise, symptom-selected composite mental health score and its threshold further aligned with these findings and may help prioritize targeted screening. Collectively, these results underscore the relevance of assessing both partners’ mental health and considering socioeconomic context when interpreting fertility-related findings and planning interventions.

Our partner-specific analyses showed that females with preconception depression or stress tended to take longer to conceive, consistent with findings reported in previous studies (Lynch *et al.*, 2014; Wesselink *et al.*, 2018; Liao *et al.*, 2024). In contrast to some earlier research (Liao *et al.*, 2024), we did not observe a significant association between male depression and infertility, a discrepancy possibly attributable to differences in study populations and sample sizes. Importantly, although depression was not significantly related to infertility, the significant association observed for anxiety may reflect the frequent comorbidity between the two conditions (Nillni *et al.*, 2016). Additionally, several studies have found no significant association between male stress and infertility, which is consistent with our findings (Wesselink *et al.*, 2018; Carter *et al.*, 2023).

In the couple-based analysis, the results showed that stress in both partners was associated with infertility, suggesting that interactions between couples may be related to a higher risk of infertility. Wesselink *et al.* (2018) have identified possible mechanisms by which stress may disrupt the pulsatile secretion of LH and testosterone, thereby impairing spermatogenesis and sperm quality (Gollenberg *et al.*, 2010; Corona *et al.*, 2016). In females, higher α-amylase levels are associated with lower fecundability. This may be due to high stress levels causing a delay or suppression of the LH surge, stress-induced alterations in oocyte transport, or changes in autoimmune status that are unfavourable for implantation (Schenker *et al.*, 1992; Ferin, 1999; Makrigiannakis *et al.*, 2001). Thus, when both partners simultaneously experience elevated stress levels, a higher risk of infertility was observed.

Using LPA, we observed that depression, anxiety, and stress were highly correlated within individuals of both sexes, indicating that these psychological symptoms typically occurred concurrently, with few individuals experiencing high levels of one symptom but low levels of the others (Yang *et al.*, 2023; Cathomas *et al.*, 2024; Hu *et al.*, 2025). Our LPA findings further highlight the need to examine couples as a unit, given the observed variability in psychological profiles between partners and the distinct associations of these combined profiles with couple fecundability. To our knowledge, no previous study has investigated the specific couple-level patterns of psychological profiles in relation to infertility. Only one study reported that discordant stress levels between partners may be associated with a higher risk of infertility (Wesselink *et al.*, 2018).

Interestingly, our findings further revealed that the decline in fecundability in the ‘F high/M medium’ group was significantly lower than that observed in the ‘F medium/M medium’ group. This finding may be explained by the notion that concordant stress levels between partners can serve as an indicator of relationship quality (Wesselink *et al.*, 2018). The ‘buffering hypothesis’ suggests that positive interpersonal relationships, particularly the couple relationship, can mitigate the negative effects of psychological problems on health (Cohen and Wills, 1985; Jackson, 1992). Thus, if relationship quality influences the association between psychological health and fecundability, and discordant psychological profiles between partners indicate poorer relationship quality, this could explain the observed group difference (Cohen and Wills, 1985; Kiecolt-Glaser and Newton, 2001).

Based on prior analyses in this research, it is clear that considering only one partner’s psychological health is insufficient. Therefore, relying on a single psychological scale to evaluate infertility risk has notable limitations. Integrating all psychological scales from both partners into a composite score may facilitate earlier intervention. After variable selection, we identified 14 symptoms that may affect fertility. Prior studies have indicated that this strategy can significantly integrate the advantages of multiple scales and is particularly suitable for specific subfields (Clarke and Kuhl, 2014; Pelham *et al.*, 2019). Our AF analysis indicated that over 8% of the lower fecundability was attributed to couples’ composite mental health scores. Up to 46%, 17% and 21% lower fecundability were attributable to poor mental health composite scores among males, females, and couples with both partners having lower education levels, respectively. Considering the significant role of SES in fecundability, SES may synergize with mental health to lower fecundability (Jørgensen *et al.*, 2023). In contrast, individual scales of depression, anxiety, or stress did not achieve statistical significance. Therefore, clinical screening of reproductive-aged couples should incorporate this novel composite scale to facilitate early intervention and potentially decrease infertility incidence, particularly among males and females with lower education levels.

Our study highlights the significant role of SES in the association between couples’ mental health and fertility. Specifically, we observed that the effects of poor mental health on fertility were more pronounced in lower SES couples. This finding aligns with previous research demonstrating that individuals with lower SES are more vulnerable to mental health challenges, which can, in turn, affect reproductive outcomes (Baum *et al.*, 1999; Flaskerud *et al.*, 2012). The mechanisms behind this may include limited access to mental health resources, lower health literacy, and greater exposure to stressors among individuals with lower SES (Mitchell and Christian, 2019; Stepanous *et al.*, 2023). These factors can worsen the negative impact of mental health conditions on reproductive health. Moreover, couples with lower SES may face additional barriers to accessing timely and effective mental health care, further influencing their fertility outcomes (Williamson *et al.*, 2019).

Our findings underscore the need to consider SES when assessing and managing couples’ mental health in fertility care. In practice, services could pilot brief couple-based screening with validated tools alongside our fertility-targeted mental health composite score to identify higher-risk couples, followed by stepped support (e.g. psychoeducation (Cousineau *et al.*, 2008; Heredia *et al.*, 2020) and positive psychological intervention (Zhang *et al.*, 2017; Dube *et al.*, 2023)). Implementation should be explicitly equity-focused, with attention to accessible delivery, cultural, and linguistic adaptation (Gameiro *et al.*, 2019; Kirubarajan *et al.*, 2021). A critical appraisal of utility is also warranted: the evidence is observational and non-causal, leaving room for residual confounding and measurement error. The composite score requires external validation (discrimination, calibration, transportability), careful threshold setting, and monitoring for potential harms (false positives, stigma, privacy) (Yokota *et al.*, 2022). Ultimately, pragmatic trials are needed to determine whether targeted, couple-focused screening and support improve fertility and whether these approaches are acceptable, scalable, and cost-effective (Patel *et al.*, 2018; Untaaveesup *et al.*, 2024).

Although the possibility of reverse causality, in which subfertility may cause mental health conditions, cannot be completely excluded, our findings provide multiple lines of evidence that do not support this explanation. The mental health assessments were conducted prior to conception attempts, and participants were unaware of their future fertility status at baseline, ensuring the temporal precedence of exposure. More importantly, significant associations between poor mental health and lower fertility were observed only among couples with lower SES, while no such associations were found in the higher SES group. If reverse causality were the primary driver, comparable patterns would be expected across SES strata, particularly given the greater fertility awareness and healthcare access in higher SES populations (Swift and Liu, 2014; Hoffman *et al.*, 2020; Imrie *et al.*, 2023). Furthermore, our sensitivity analyses showed that the observed associations remained robust when using both subfecundability and subfertility as outcome definitions. Additionally, Mendelian randomization studies have demonstrated a causal effect of major depressive disorder on female infertility, with interleukin-18 acting as a potential mediator of this pathway (Li *et al.*, 2024; Mao *et al.*, 2024). Together, these findings support a directional effect of preconception mental health on reproductive outcomes and strengthen the internal validity of our conclusions.

### Strengths and limitations

Our study has both strengths and limitations. Our prospective study provides a clear temporal relationship between exposure and outcome. We collected demographic information and assessed the mental health of both male and female partners. We conducted the q-gcomp to assess the mix effect and fitted distinct patterns of couples’ mental health by using LPA for the first time. Our large sample size provided high statistical power to assess the associations. Multiple statistical models and sensitivity analyses were employed to achieve balanced and robust conclusions. On the other hand, our study has several limitations. First, pregnancy and couples’ mental health were assessed based on self-reported data rather than through clinical tests like the human chorionic gonadotropin test and α‐amylase test, which could have provided a more accurate diagnosis. Second, including females with irregular menstrual cycles might have introduced bias, as our method for calculating TTP is more dependable for those with regular cycles. Third, although the possibility of reverse causality cannot be completely excluded, the temporal order of the assessment and the SES-stratified results alleviate the likelihood of this explanation. Fourth, despite our efforts to adjust for various confounding variables, some factors, such as the use of medications (notably antidepressants), the frequency of sexual intercourse and previous depressive and anxious symptoms, could still be affecting our results. Fifth, because occupational classifications are subjective and culturally specific (Jespersen *et al.*, 2025), and there is currently no appropriate formula in China to construct a composite SES index, we used education level and family income as alternative indicators of SES. This strategy is consistent with most Chinese epidemiological studies (Wang *et al.*, 2014; Rosengren *et al.*, 2019; Li *et al.*, 2021), more broadly, with international practice (An *et al.*, 2020; Jespersen *et al.*, 2025), where education and income are widely used, given their higher validity (Zhu *et al.*, 2023; Wang *et al.*, 2024). Finally, due to the small sample size after stratification, the effect modification by SES could not be examined in the couple-based models. Nevertheless, this limitation was partly overcome by our use of LPA, which captured heterogeneous mental health patterns within couples and provided complementary insights.

## Conclusions

Our findings show that poorer mental health in either partner was associated with lower fertility, and the association was stronger when both partners reported problems. The adverse impact of mental health on fertility was more pronounced among couples with lower SES, emphasizing the modifying role of education and income in this association. These findings highlight that preconception care should include mental health assessment and support for both partners, not just women. Integrating couple-based mental health care into preconception services may help improve fertility outcomes and reduce disparities, particularly among couples of low SES. Further research is warranted to elucidate the mechanisms linking mental health, SES, and fertility.

## Supplementary Material

hoaf071_Supplementary_Data

## Data Availability

The data underlying this article will be shared on reasonable request to the corresponding author.
