# Supplementary material for: Mental health of couples affects fertility, modified by socioeconomic status: a couple-based analysis
Source: Hum Reprod Open. 2025 Nov 14;2025(4):hoaf071. doi: 10.1093/hropen/hoaf071 (PMC12976685; doi:10.1093/hropen/hoaf071)
Supplement: hoaf071_Supplementary_Data [file hoaf071_Supplementary_Data.docx]

**Supplemental Online Content**

**Supplementary Materials and methods.** Statistical methods for attributable fraction estimation.

**Supplementary Figure S1.** Study flow chart.

**Supplementary Figure S2.** Directed acyclic graph for the associations between couples’ mental health and fecundability.

**Supplementary Figure S3.** Exposure-response curves between a couples’ mental scores and couples’ fecundability.

**Supplementary Figure S4.** Exposure-response curves between a couples’ mental scores and couples’ infertility.

**Supplementary Figure S5.** Distinct patterns of couples’ depression, anxiety, and stress Z-score.

**Supplementary Figure S6.** Multiple-exposure for the associations between couples’ mental health subdomain exposures to fecundability in the elastic net regression (A and B; α = 0.1, λ = 0.389).

**Supplementary Figure S7.** An optimal cut-off value of the couples’ mental health composite score in fecundability.

**Supplementary Figure S8.** Time-dependent attributable fraction of fecundability in the couples’ mental health composite score.

**Supplementary Figure S9.** Time-dependent attributable fraction of fecundability in couples’ depression, anxiety, and stress.

**Supplementary Figure S10.** Educational subgroup analyses of time-dependent attributable fraction of fecundability in the couples’ mental health composite score.

**Supplementary Figure S11.** Economic subgroup analyses of time-dependent attributable fraction of fecundability in the couples’ mental health composite score.

**Supplementary Figure S12.** Couples’ educational subgroup analyses of time-dependent attributable fraction of fecundability in the couples’ mental health composite score.

**Supplementary Figure S13.** Time-dependent attributable fraction of fecundability in the couples’ mental health composite score with imputed data.

**Supplementary Figure S14.** Time-dependent attributable fraction of subfecundability in the couples’ mental health composite score.

**Supplementary Figure S15.** Stratified analyses of a time-dependent attributable fraction of fecundability in the couples’ mental health composite score.

**Supplementary Table S1.** Educational subgroup analyses of the association between couples’ mental health mixture and infertility (q-gcomp model).

**Supplementary Table S2.** Financial subgroup analyses of the association between couples’ mental health mixture and infertility (q-gcomp model).

**Supplementary Table S3.** Couples’ educational subgroup analyses of the association between couples’ mental health mixture and infertility (q-gcomp model).

**Supplementary Table S4.** Latent profile analysis model summary table.

**Supplementary Table S5.** Association of distinct couples’ mental health scores with their fecundability.

**Supplementary Table S6.** Association of distinct couples’ mental health scores with their fertility in different females’ educational subgroups.

**Supplementary Table S7.** Association of distinct couples’ mental health scores with their fertility in different males’ educational subgroups.

**Supplementary Table S8.** Association of distinct couples’ mental health scores with their fertility in different financial subgroups.

**Supplementary Table S9.** Association of distinct couples’ mental health scores with their fertility in different couples’ educational subgroups.

**Supplementary Table S10.** Multiple-exposure coefficient for the associations between couples’ mental health subdomain exposures to fecundability in elastic net regression (α = 0.1, λ = 0.389).

**Supplementary Table S11.** Attributable fraction of infertility caused by couples’ mental health.

**Supplementary Table S12.** Educational subgroup analyses of time-dependent attributable fraction of infertility in the couples’ mental health composite score.

**Supplementary Table S13.** Financial subgroup analyses of time-dependent attributable fraction of infertility in the couples’ mental health composite score.

**Supplementary Table S14.** Couples’ educational subgroup analyses of time-dependent attributable fraction of infertility in the couples’ mental health composite score.

**Supplementary Table S15.** Associations between each partner’s mental health and couple fecundability with imputed data.

**Supplementary Table S16.** Associations between each partner’s mental health and couple subfecundability and subfertility.

**Supplementary Table S17.** Associations between each partner’s mental health and their fecundability in females with regular menstrual cycle and nulliparous couples.

**Supplementary Table S18.** Associations between couples’ depression, anxiety, and stress and their fecundability with data imputation.

**Supplementary Table S19.** Associations of couples’ depression, anxiety, and stress with their subfecundability and subfertility.

**Supplementary Table S20.** Associations of couples’ depression, anxiety, and stress with their fecundability in females with regular menstrual cycle and nulliparous couples.

**Supplementary Table S21.** Association of distinct couples’ mental health scores with their fertility with data imputation.

**Supplementary Table S22.** Association of distinct couples’ mental health scores with their subfecundability and subfertility.

**Supplementary Table S23.** Association of distinct couples’ mental health scores with their fertility in females with regular menstrual cycle and nulliparous couples.

**Supplementary Table S24.** Attributable fraction of infertility caused by couples’ mental health with data imputation.

**Supplementary Table S25.** Attributable fraction of subfertility caused by couples’ mental health.

**Supplementary Table S26.** Attributable fraction of infertility caused by couples’ mental health in females with regular menstrual cycle and nulliparous couples.

**Supplementary Materials and methods. Statistical methods for attributable fraction estimation.**

In our analyses, we calculated the attributable fraction (AF) for two types of outcomes: a time-to-event outcome based on TTP and pregnancy (AFcoxph), and a binary outcome based on infertility (AFglm). For the AFcoxph, the AF was estimated under the hypothetical scenario in which a binary exposure $X$ is eliminated from the population. The estimate was adjusted for confounders $Z$ using the Cox proportional hazards model. Let the AF function be defined as:

$$AF=1-\frac{\{1-S_{0}(t)\}}{\{1-S(t)\}}$$

Where $S_{0}(t)$ denotes the counterfactual survival function for the event if the exposure would have been eliminated from the population at baseline and $S(t)$ denotes the factual survival function. If $Z$ is sufficient for confounding control, then $S_{0}(t)$ can be expressed as $\begin{matrix} E_{Z}\{S(t|X=0,Z)\} \end{matrix}$. The function uses a fitted Cox proportional hazards regression to estimate $S(t|X=0,Z)$, and the marginal sample distribution of $Z$ to approximate the outer expectation (Biometrika. 2010 Sep;97(3):713-726; Stat Methods Med Res. 2017 Apr;26(2):948-969.).

AFglm estimates the attributable fraction for a binary outcome $Y$ under the hypothetical scenario where a binary exposure $X$ is eliminated from the population. The estimate is adjusted for confounders $Z$ by logistic regression. AFglm can be defined as:

$$AF=1-\frac{Pr(Y_{0}=1)}{Pr(Y=1)}$$

where $Pr(Y_{0}=1)$ denotes the counterfactual probability of the outcome if the exposure would have been eliminated from the population and $Pr(Y=1)$ denotes the factual probability of the outcome. If $Z$ is sufficient for confounding control, then $Pr(Y_{0}=1)$ can be expressed as $E_{Z}\{Pr(Y=1|X=0,Z\}$. The function uses logistic regression to estimate $\begin{matrix} Pr(Y=1|X=0,Z) \end{matrix}$, and the marginal sample distribution of $Z$ to approximate the outer expectation (Biometrics. 1993 Sep;49(3):865-72; Biostatistics. 2011 Jan;12(1):112-21.).


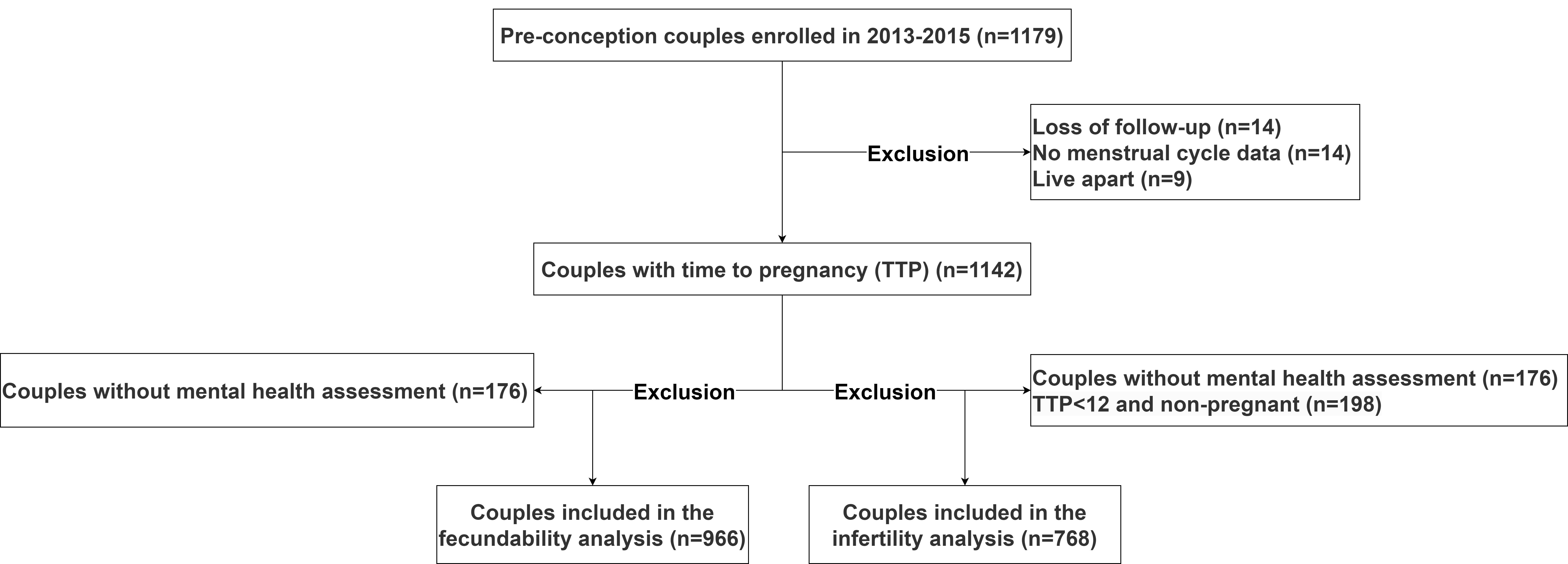


**Supplementary Figure S1. Study flow chart.**


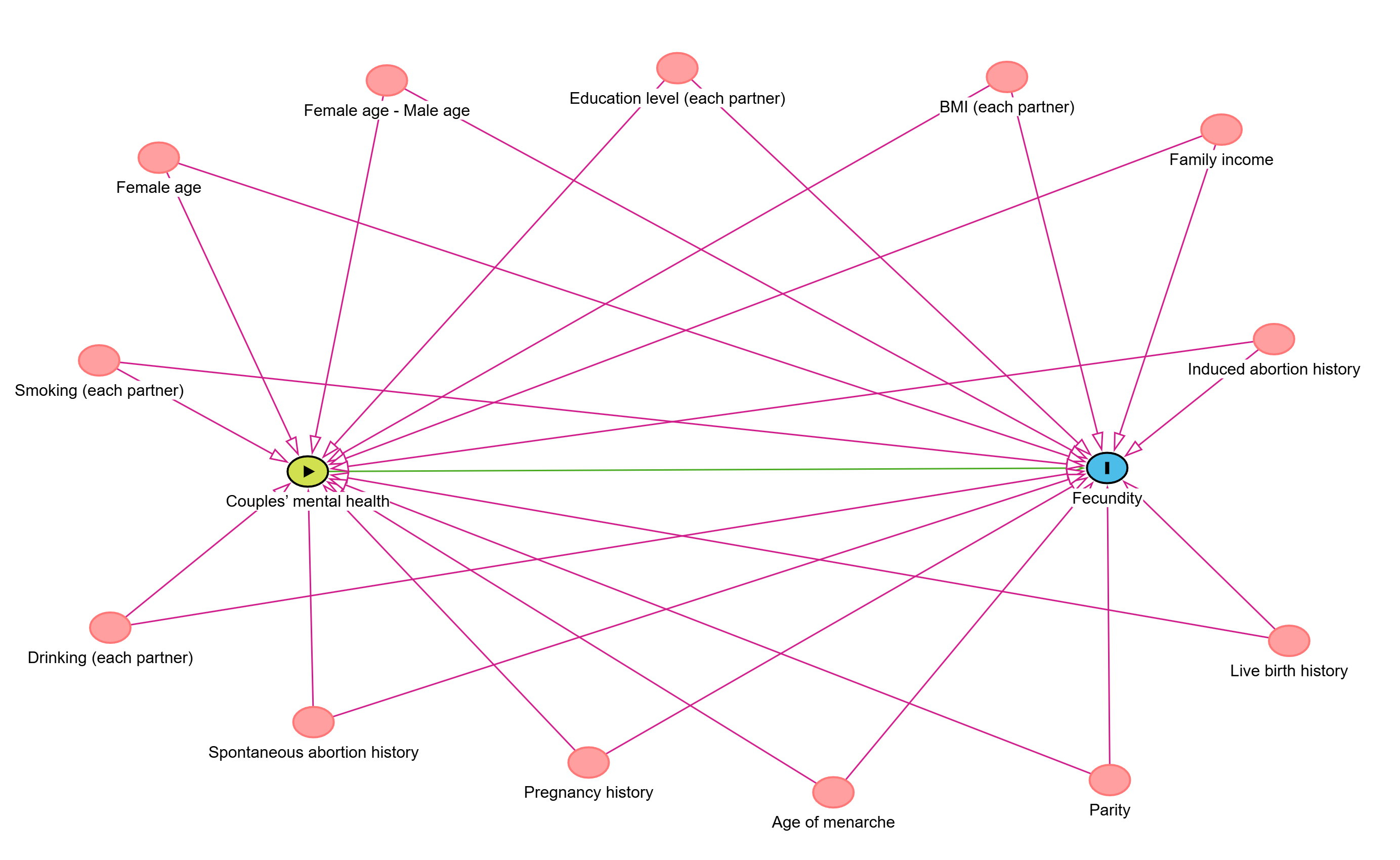


**Supplementary Figure S2. Directed acyclic graph for the associations between couples’ mental health and fecundability.**


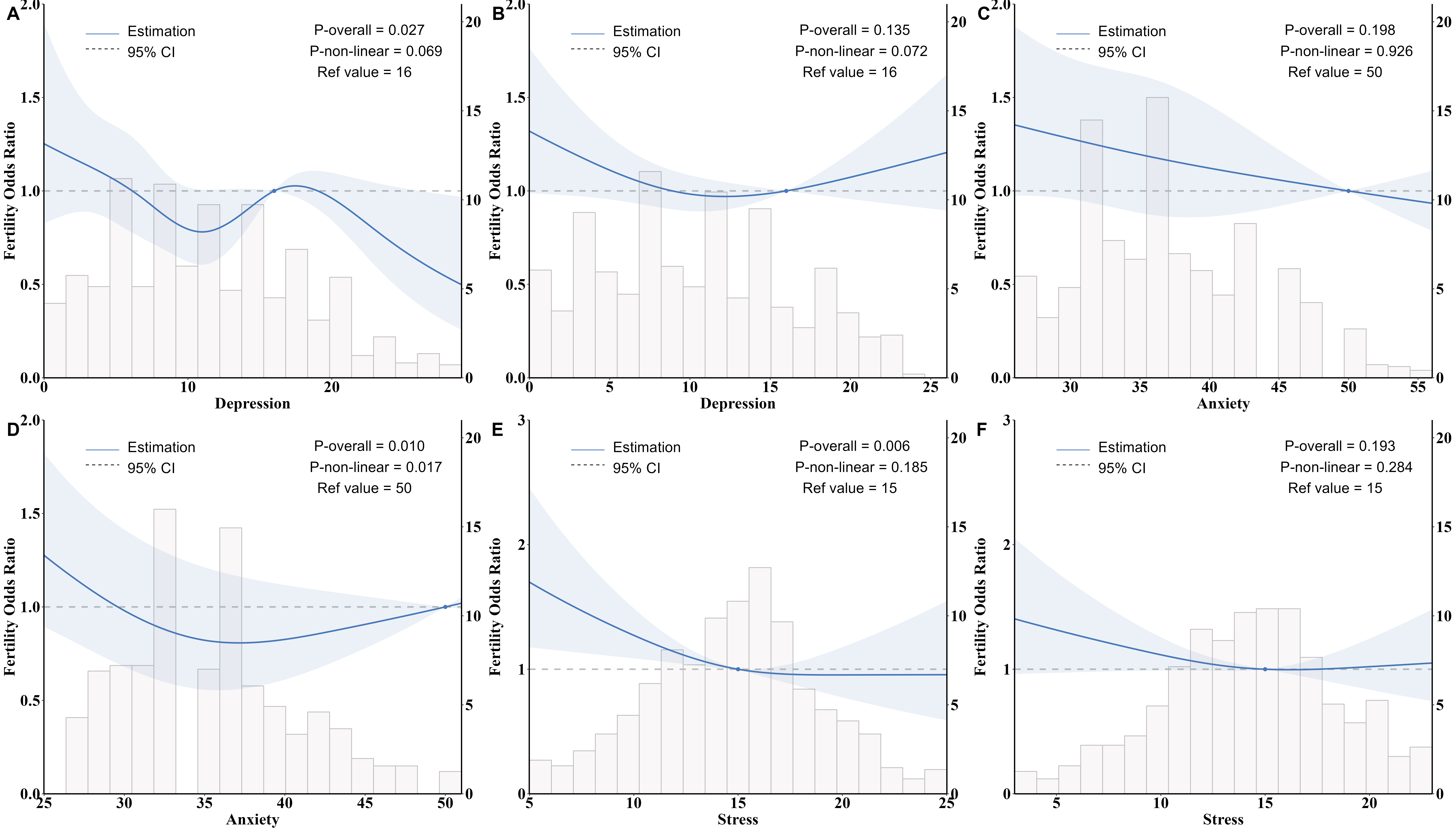


**Supplementary Figure S3. Exposure-response curves between a couples’ mental scores and couples’ fecundability.**

The areas within dashed lines indicate the 95 % CI of the predicted value. A, female depression; B, male depression; C, female anxiety; D, male anxiety; E, female stress; F, male stress. For females, data were adjusted for their age, BMI, family income, education level, smoking, drinking, age of menarche, pregnancy history, parity, live birth history, spontaneous abortion history, and induced abortion history. For males, data were adjusted for their age, BMI, family income, education level, smoking, drinking; their partner’s pregnancy history, parity, live birth history, spontaneous abortion history, and induced abortion history.


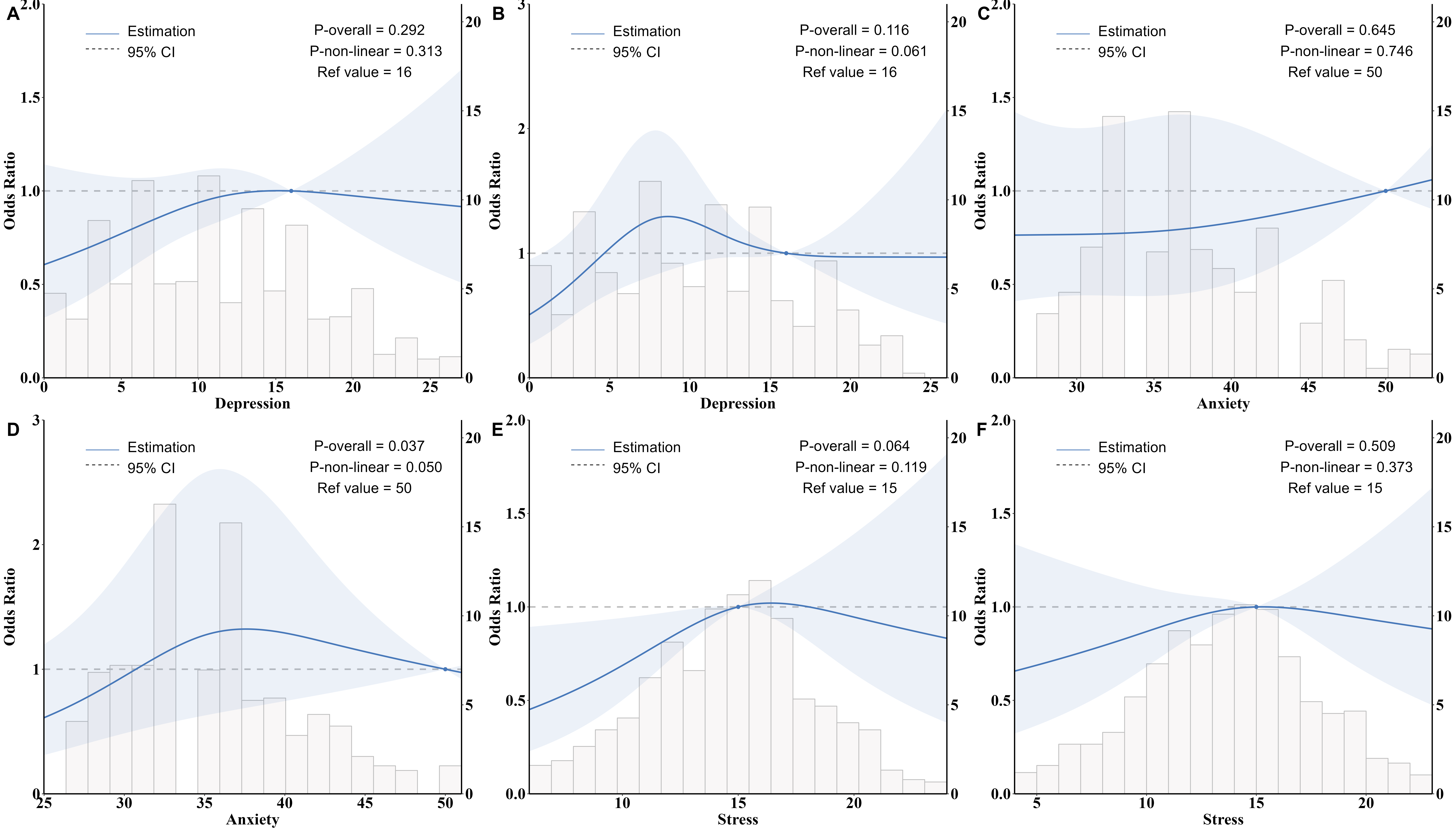


**Supplementary Figure S4. Exposure-response curves between a couples’ mental scores and couples’ infertility.**

The areas within dashed lines indicate the 95 % CI of the predicted value. A, female depression; B, male depression; C, female anxiety; D, male anxiety; E, female stress; F, male stress. For females, data were adjusted for their age, BMI, family income, education level, smoking, drinking, age of menarche, pregnancy history, parity, live birth history, spontaneous abortion history, and induced abortion history. For males, data were adjusted for their age, BMI, family income, education level, smoking, drinking; their partner’s pregnancy history, parity, live birth history, spontaneous abortion history, and induced abortion history.


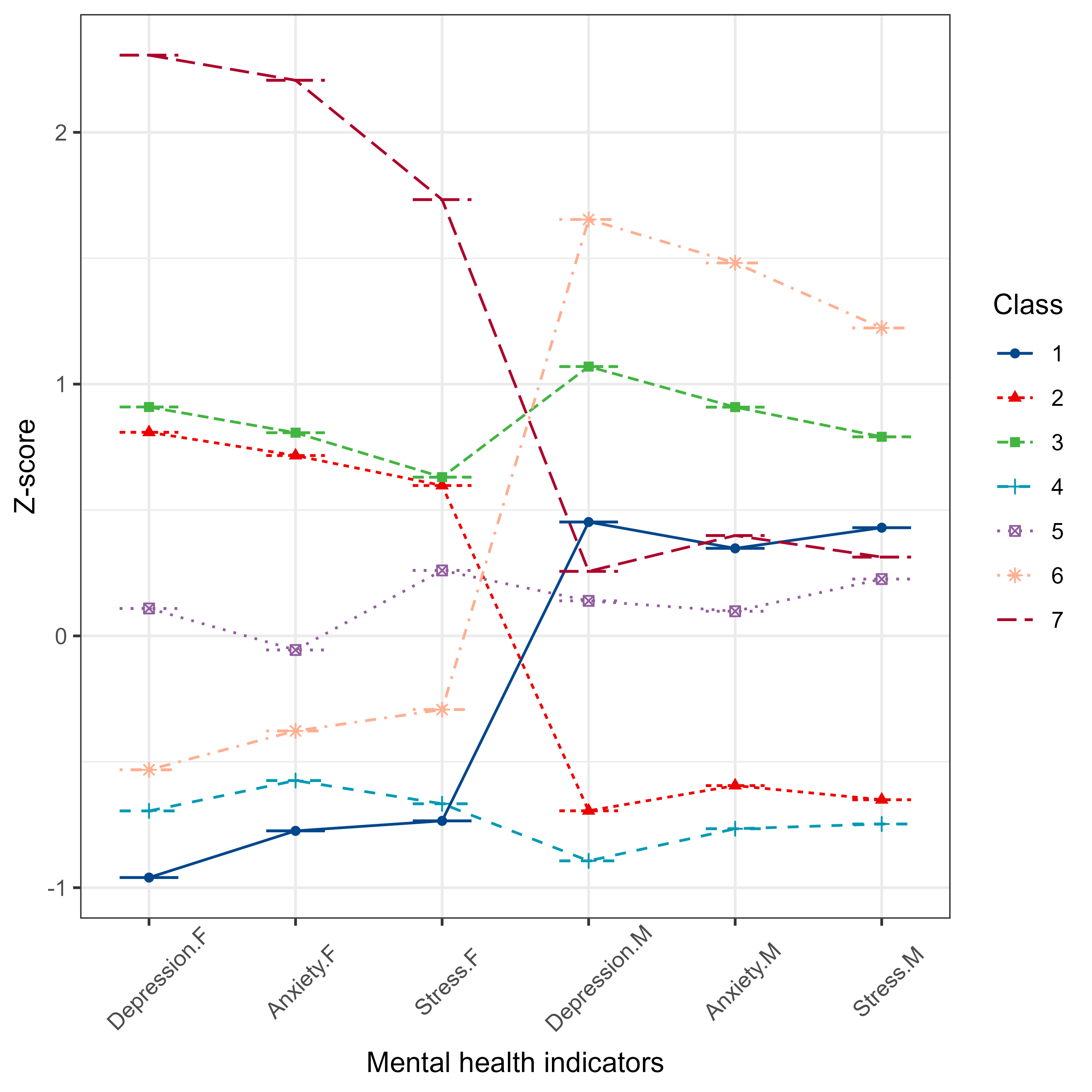


**Supplementary Figure S5. Distinct patterns of couples’ depression, anxiety, and stress Z-score.**

F, female; M, male. The horizontal axis represents the depression, anxiety, and stress scales of couples, while the vertical axis indicates the mean values of standardized scores for each scale.


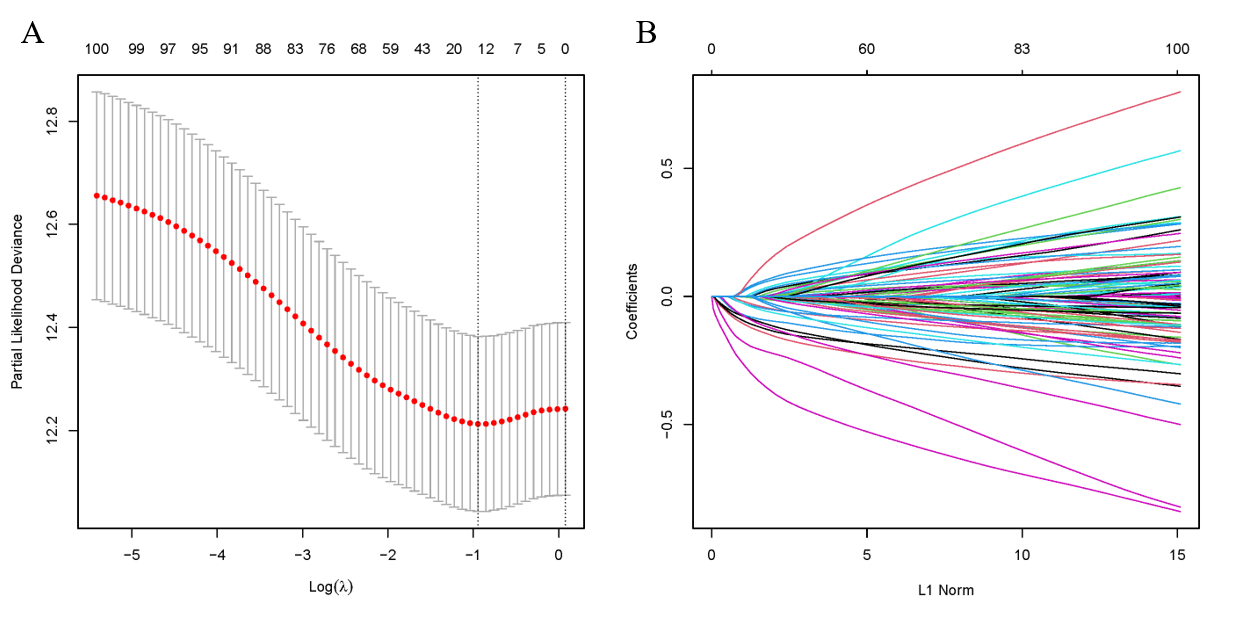


**Supplementary Figure S6. Multiple-exposure for the associations between couples’ mental health subdomain exposures to fecundability in the elastic net regression (A and B; α = 0.1, λ = 0.389).**


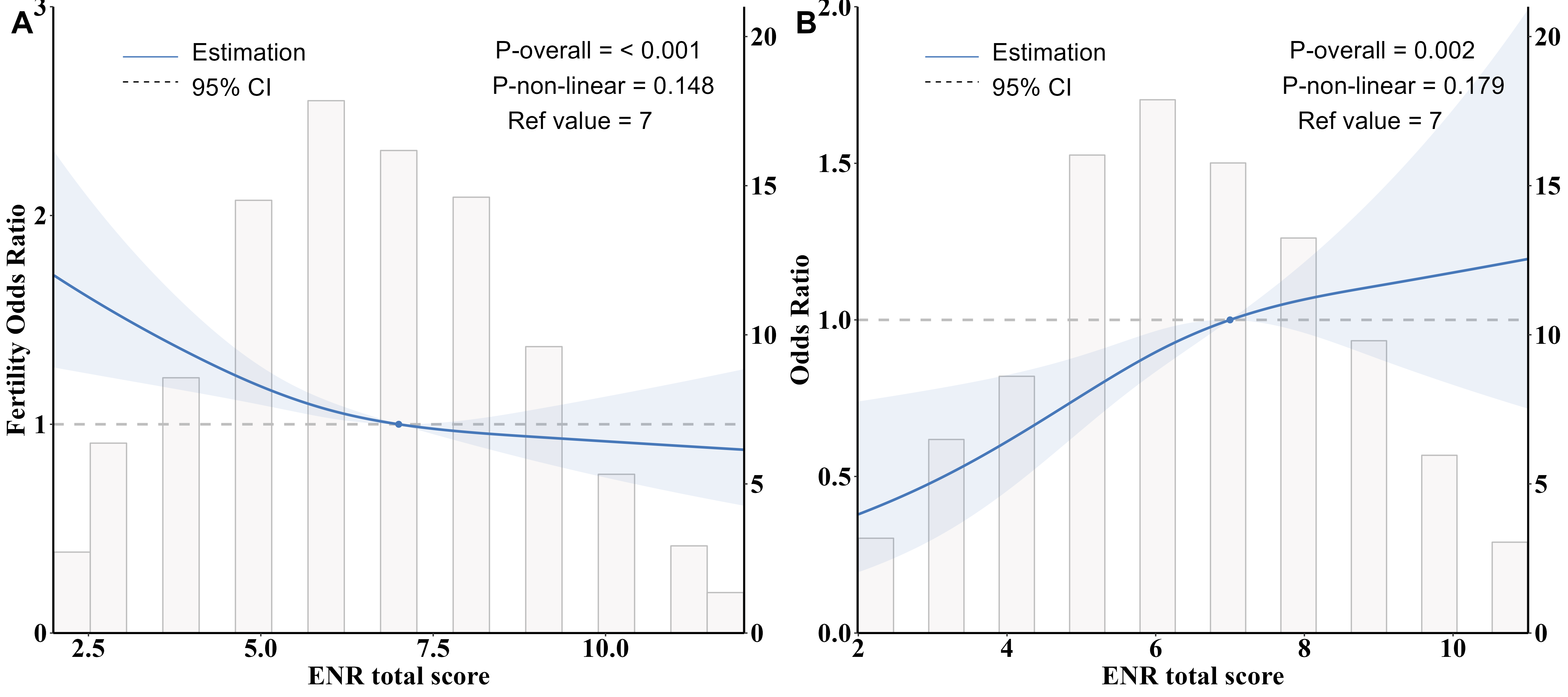


**Supplementary Figure S7. An optimal cut-off value of the couples’ mental health composite score in fecundability.**

The areas within dashed lines indicate the 95 % CI of the predicted value. A, Optimal cut-off in fecundability = 7; B, Optimal cut-off in infertility = 7.


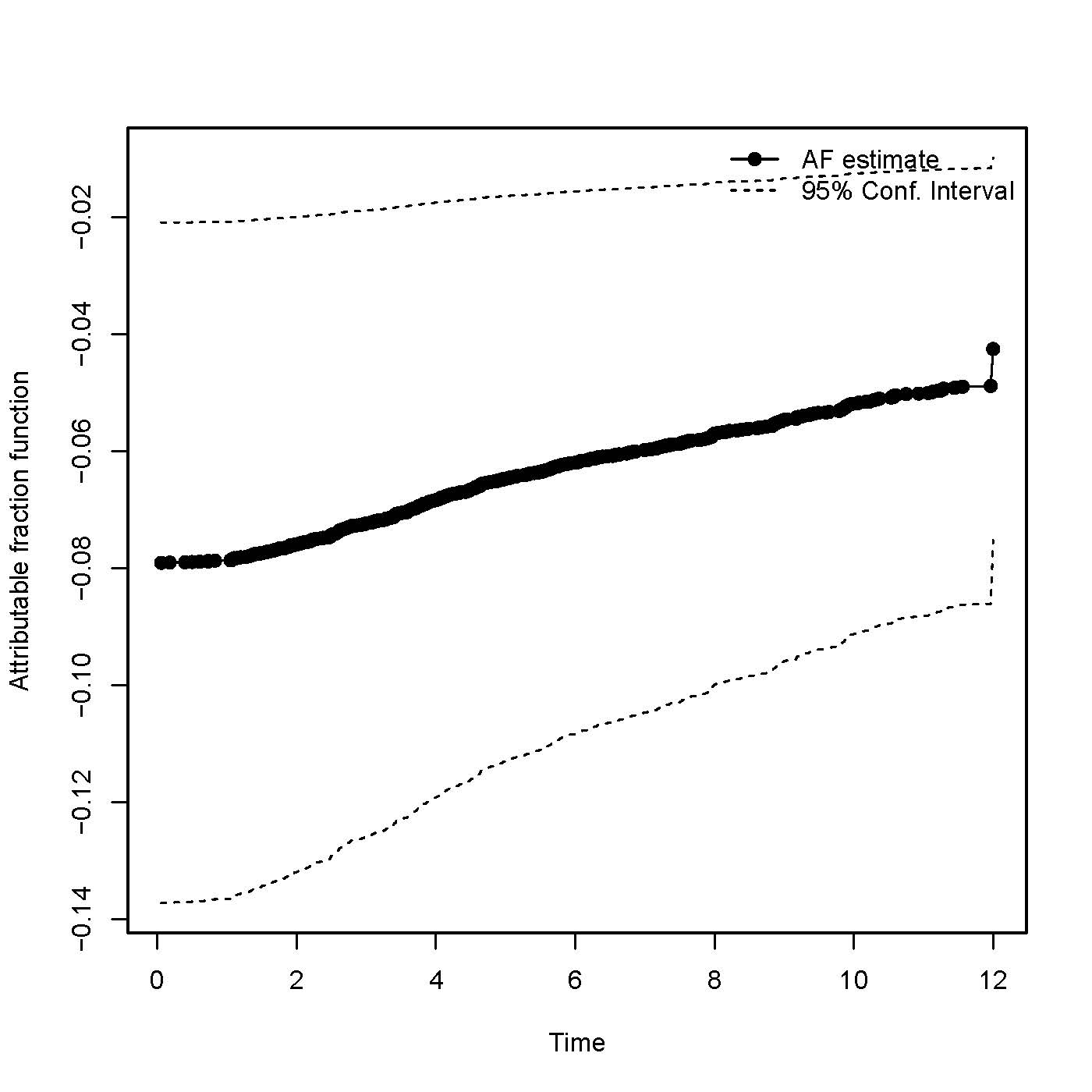


**Supplementary Figure S8. Time-dependent attributable fraction of fecundability in the couples’ mental health composite score.**

Black dots are attributable fraction at different follow-up time points. The areas within dashed lines indicate the 95 % CI of the predicted value.


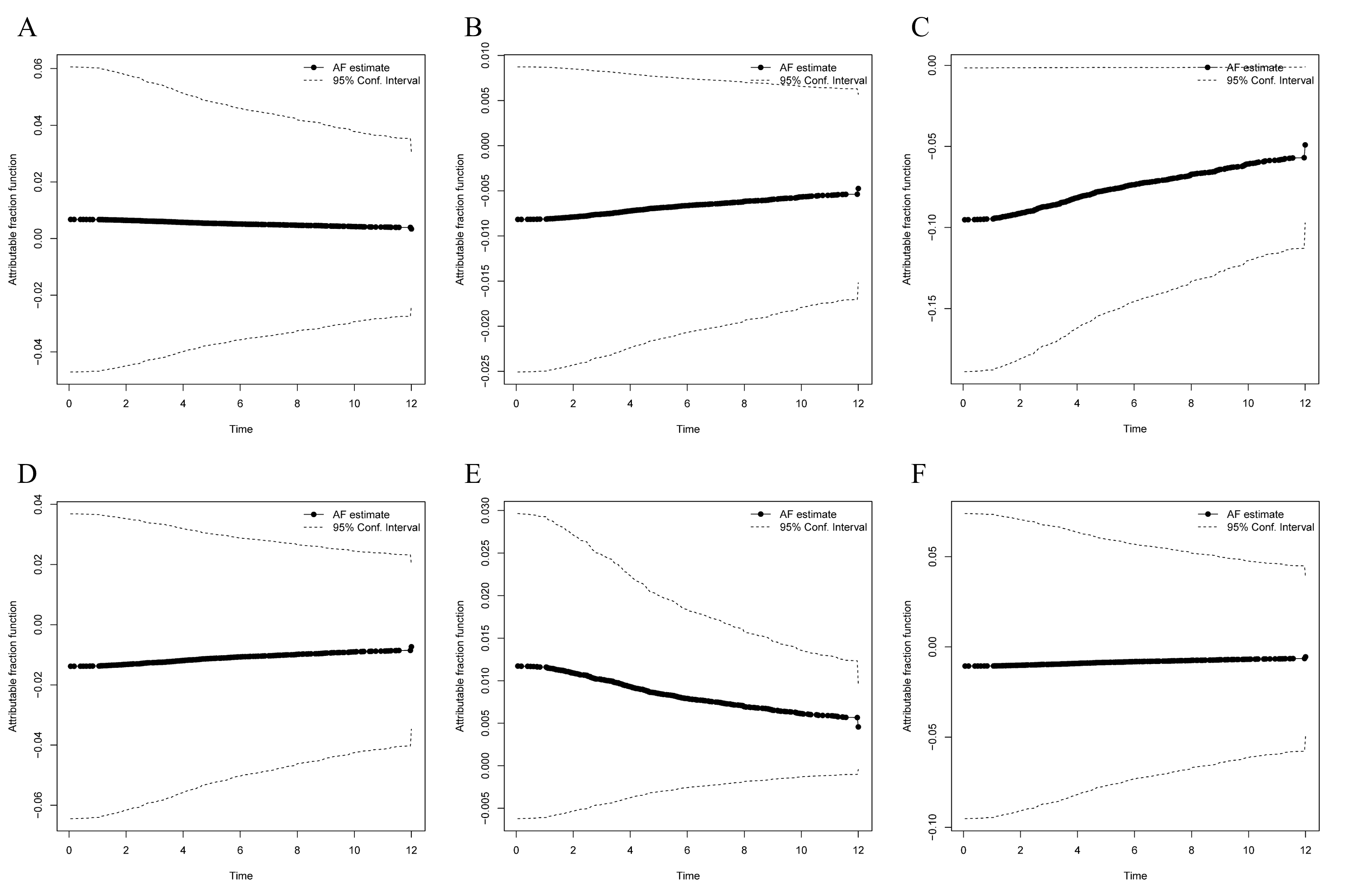


**Supplementary Figure S9. Time-dependent attributable fraction of fecundability in couples’ depression, anxiety, and stress.**

Black dots are attributable fraction at different follow-up time points. The areas within dashed lines indicate the 95 % CI of the predicted value. A, female depression; B, female anxiety; C, female stress; D, male depression; E, male anxiety; F, male stress.


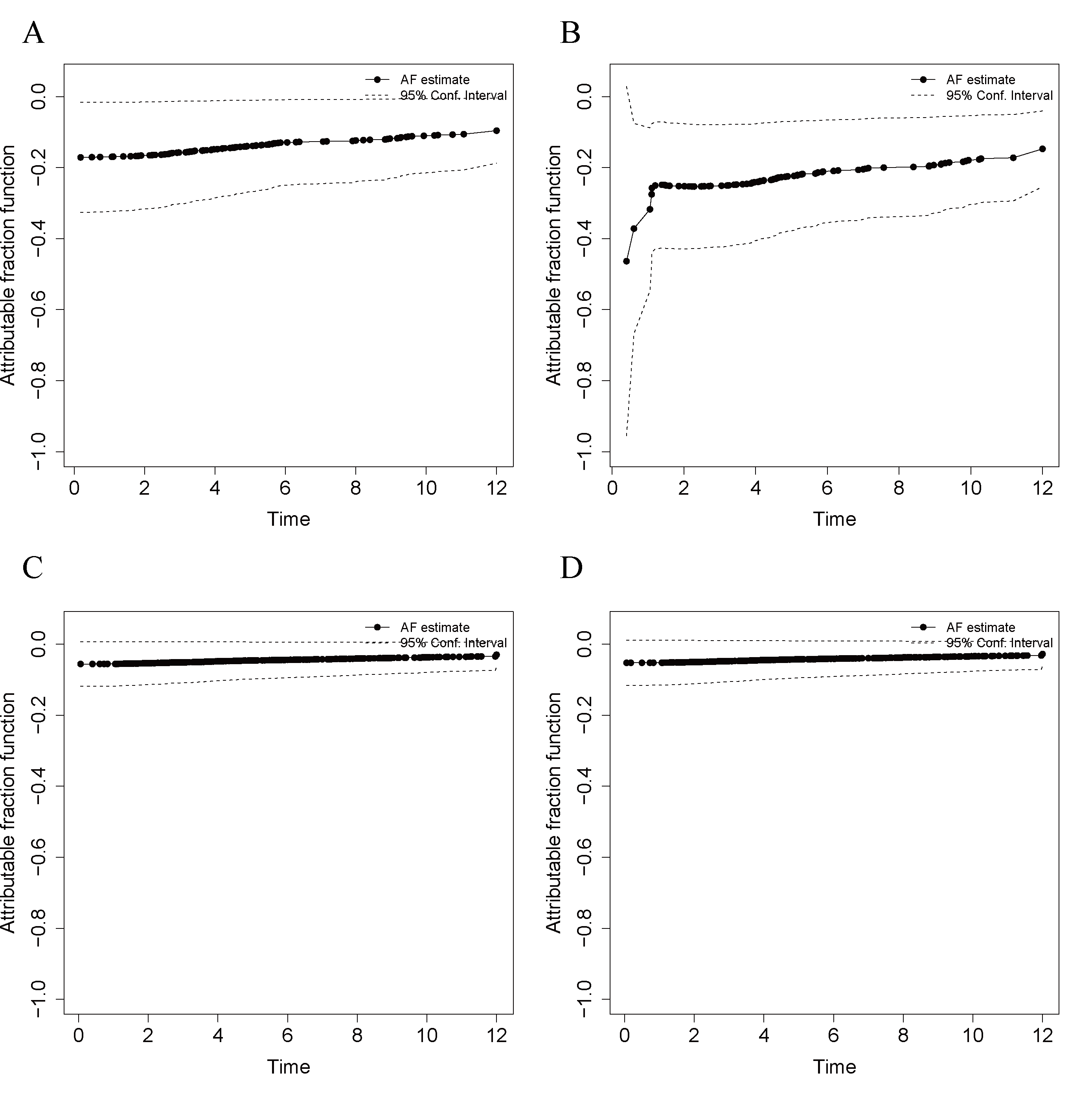


**Supplementary Figure S10. Educational subgroup analyses of time-dependent attributable fraction of fecundability in the couples’ mental health composite score.**

Black dots are attributable fraction at different follow-up time points. The areas within dashed lines indicate the 95 % CI of the predicted value. A, female < college; B, male < college; C, female ≥ college; D, male ≥ college.


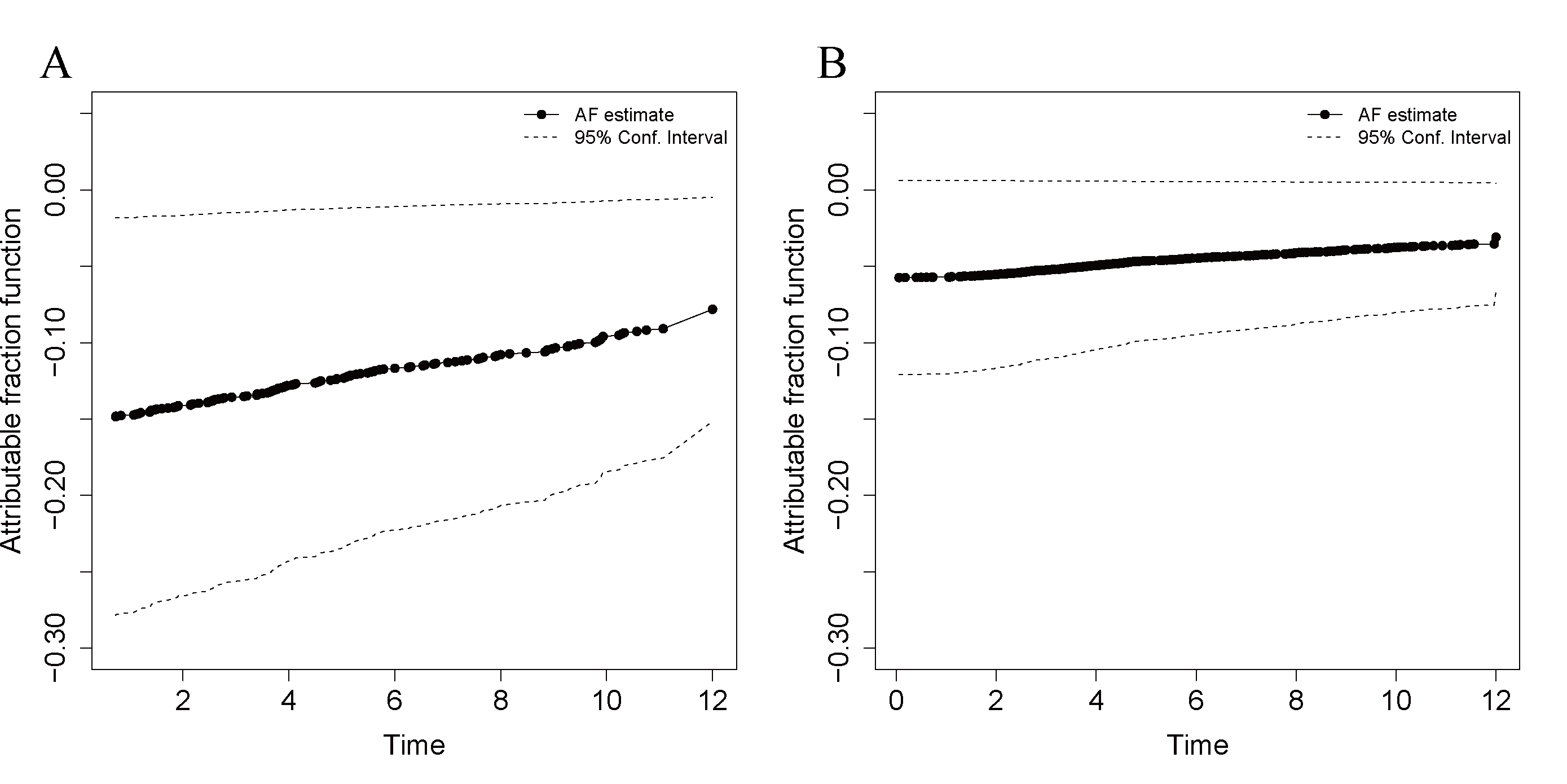


**Supplementary Figure S11. Economic subgroup analyses of time-dependent attributable fraction of fecundability in the couples’ mental health composite score.**

Black dots are attributable fraction at different follow-up time points. The areas within dashed lines indicate the 95 % CI of the predicted value. A, family income < CNY 150 000/year; B, family income ≥ CNY 150 000/year.


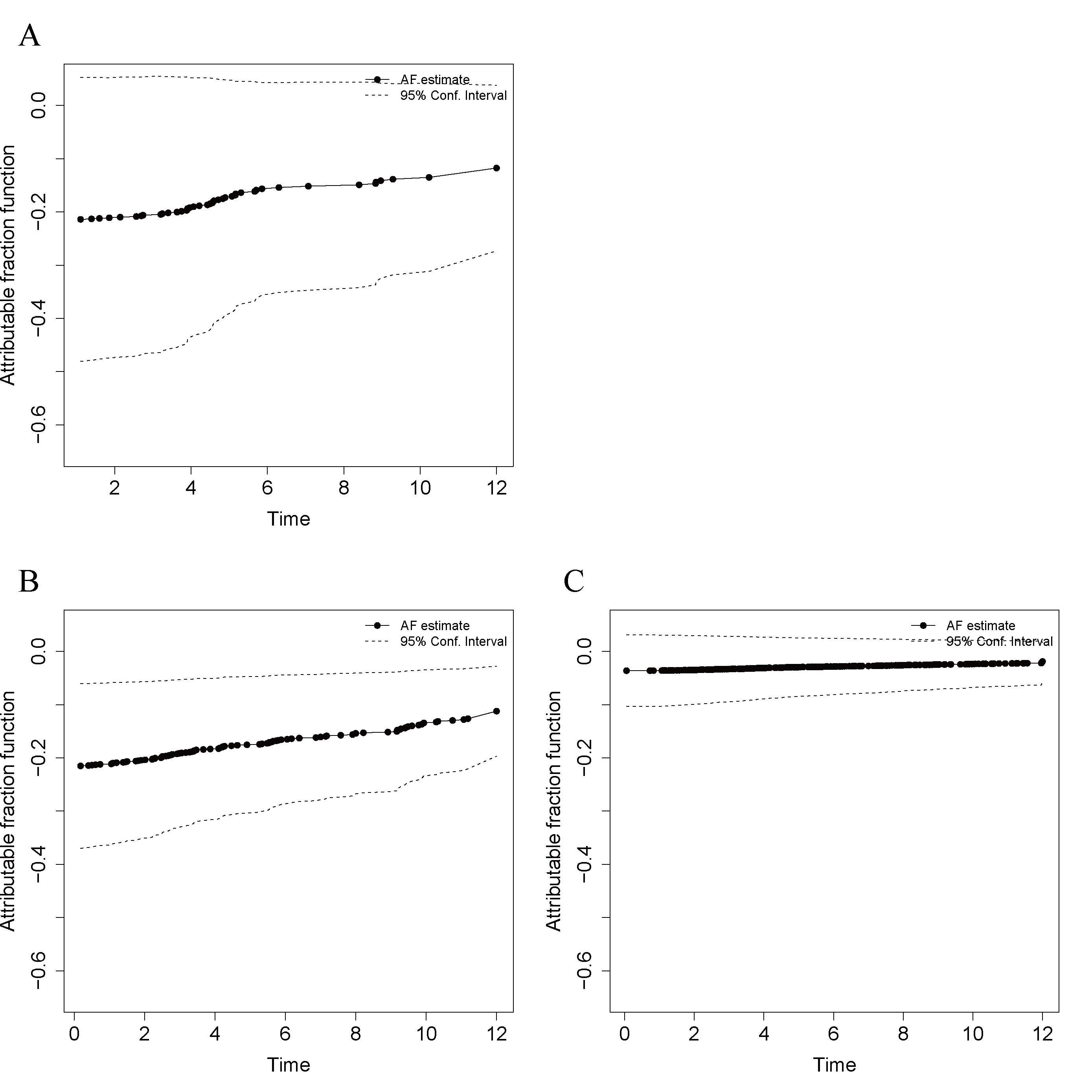


**Supplementary Figure S12. Couples’ educational subgroup analyses of time-dependent attributable fraction of fecundability in the couples’ mental health composite score.**

Black dots are attributable fraction at different follow-up time points. The areas within dashed lines indicate the 95 % CI of the predicted value. A, both < college; B, one of the couples < college; C, both ≥ college.


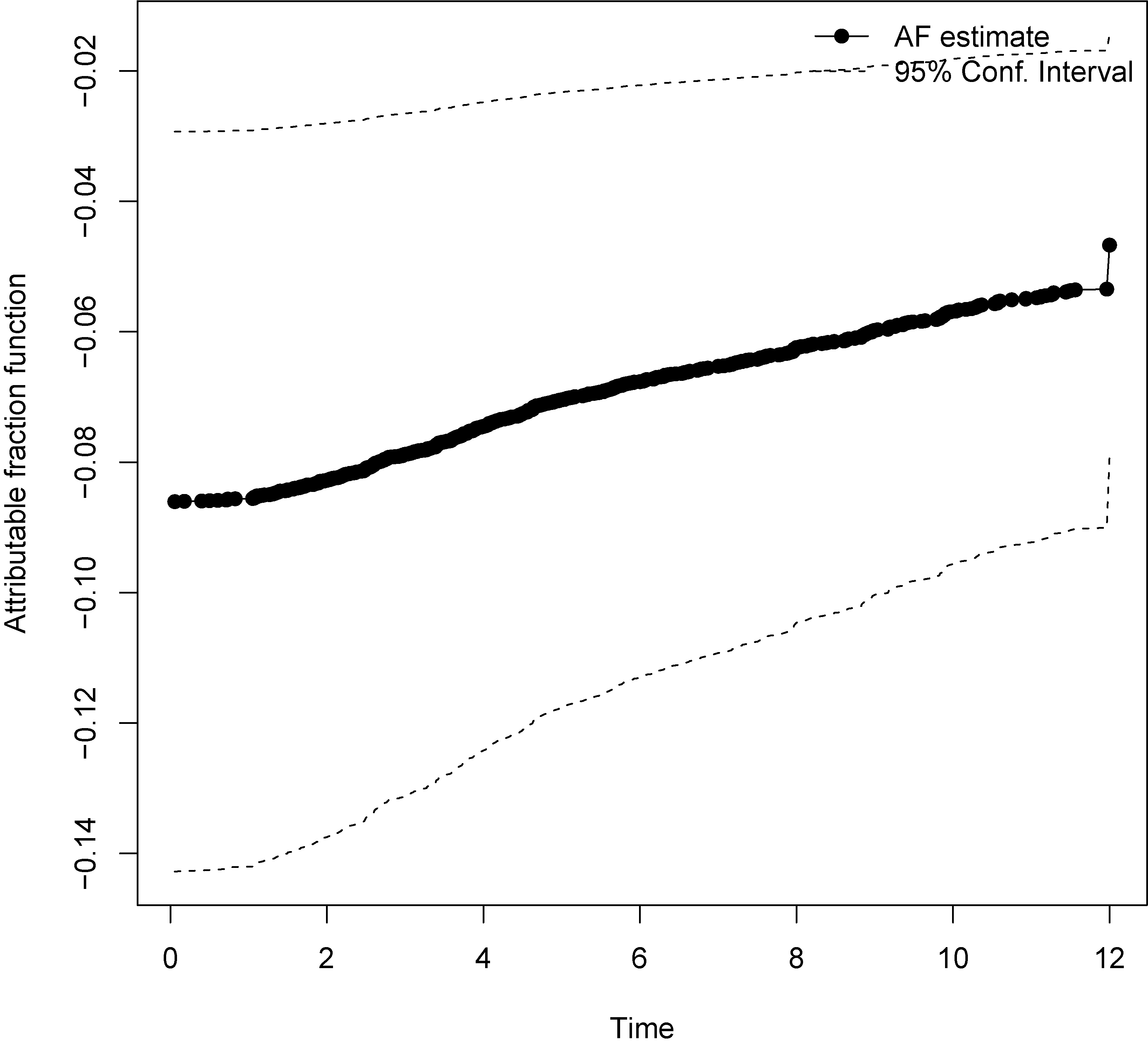


**Supplementary Figure S13. Time-dependent attributable fraction of fecundability in the couples’ mental health composite score with imputed data.**

Black dots are attributable fraction at different follow-up time points. The areas within dashed lines indicate the 95 % CI of the predicted value.


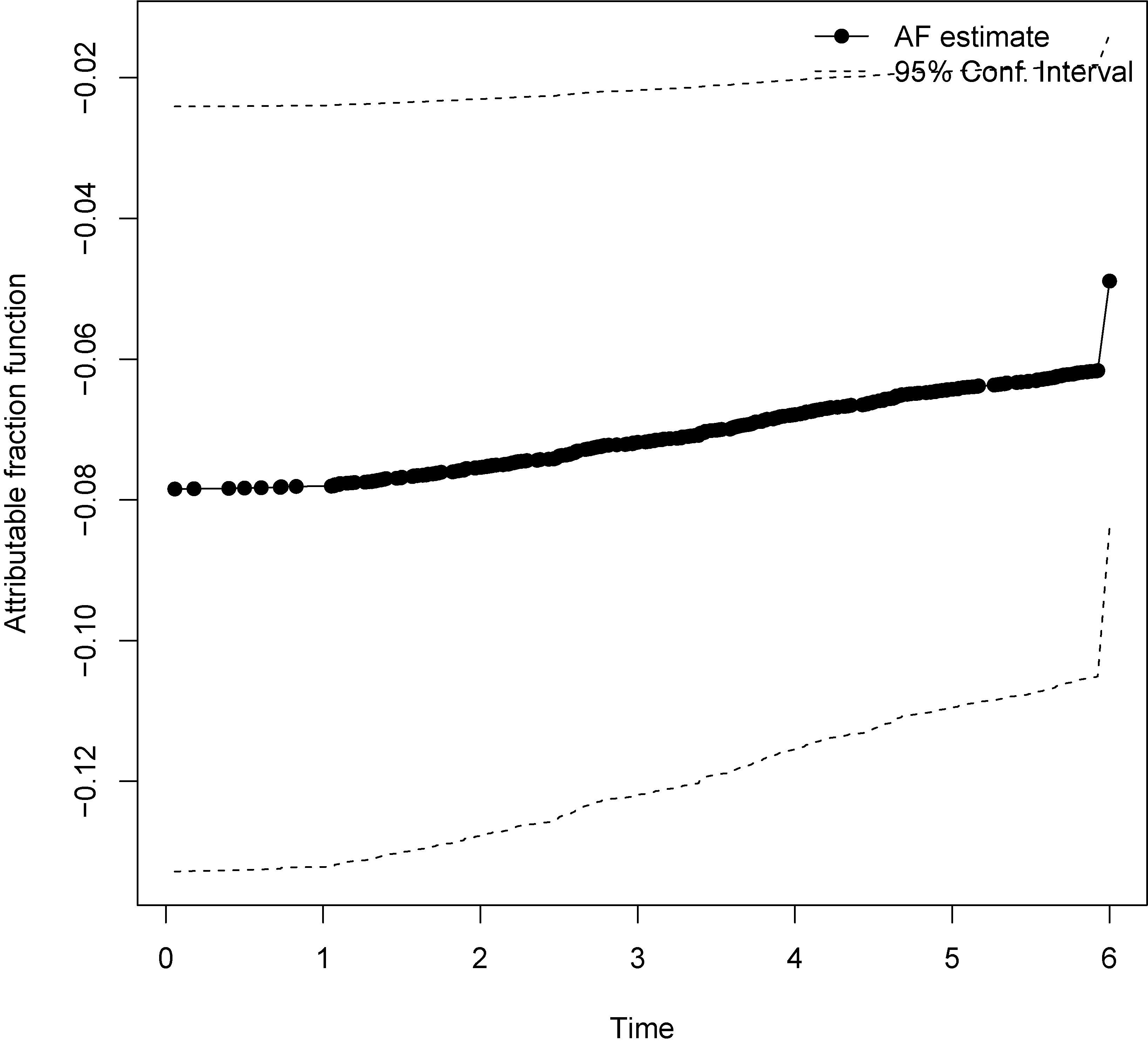


**Supplementary Figure S14. Time-dependent attributable fraction of subfecundability in the couples’ mental health composite score.**

Black dots are attributable fraction at different follow-up time points. The areas within dashed lines indicate the 95 % CI of the predicted value.


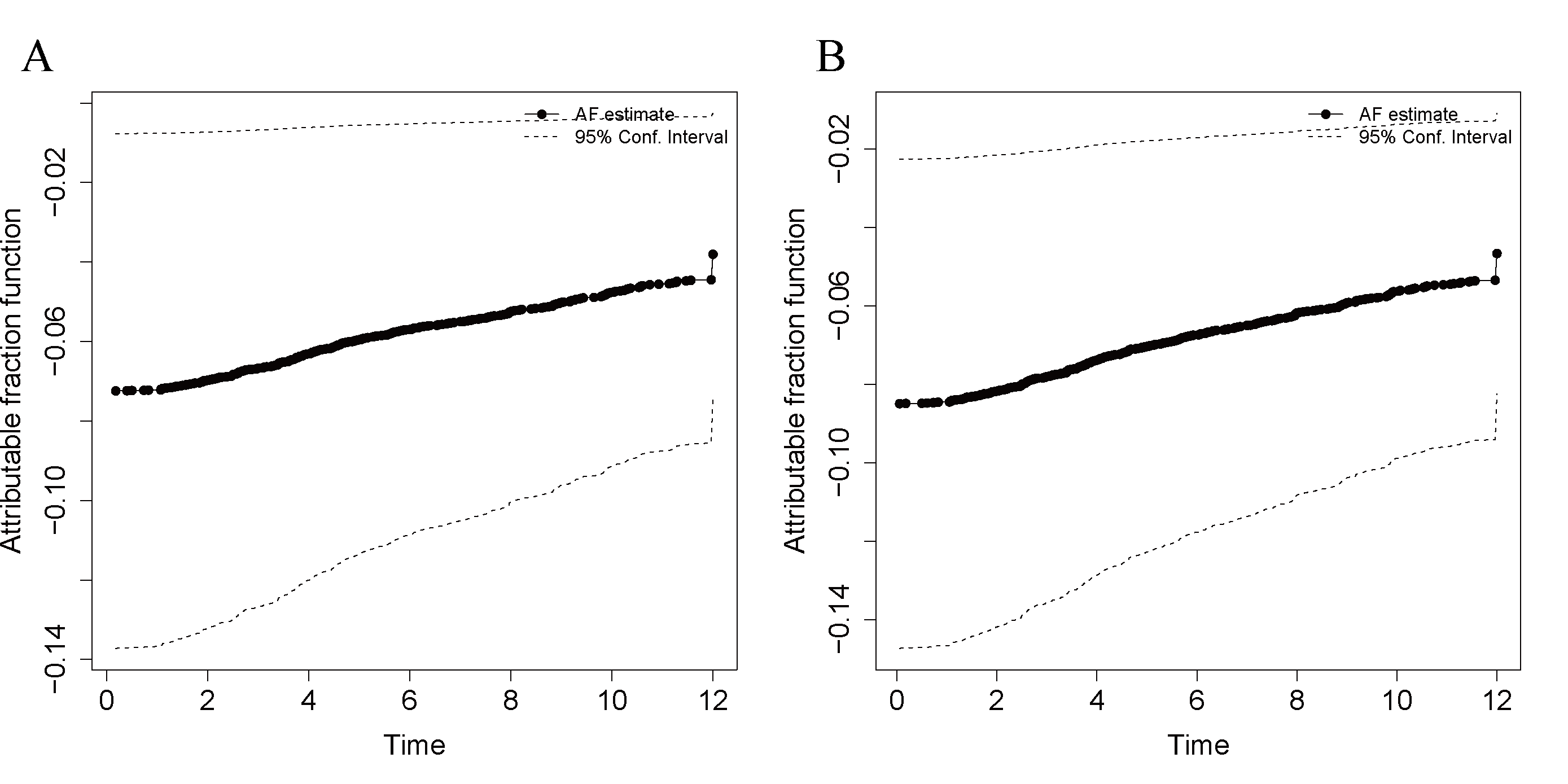


**Supplementary Figure S15. Stratified analyses of a time-dependent attributable fraction of fecundability in the couples’ mental health composite score.**

Black dots are attributable fraction at different follow-up time points. The areas within dashed lines indicate the 95 % CI of the predicted value. A, regular menstrual cycle; B, nulliparous.

**Supplementary Table S1. Educational subgroup analyses of the association between couples’ mental health mixture and infertility (q-gcomp model).**

| **Subgroup** | **< College** | **≥ College** | ***P* for interaction** |
| --- | --- | --- | --- |
| **Stratified by female education** |  |  |  |
| All mixture | 1.18 (1.05, 1.33) | 1.02 (0.96, 1.08) | 0.054 |
| Female mixture | 1.07 (0.97, 1.18) | 1.02 (0.98, 1.07) | 0.105 |
| Male mixture | 1.07 (0.99, 1.16) | 1.03 (0.99, 1.07) | 0.153 |
| Coupe depression mixture | 1.12 (1.02, 1.24) | 1.00 (0.96, 1.05) | 0.082 |
| Coupe anxiety mixture | 1.14 (1.03, 1.26) | 1.00 (0.95, 1.04) | 0.093 |
| Coupe stress mixture | 1.09 (0.99, 1.20) | 1.03 (0.98, 1.08) | 0.086 |
| **Stratified by male education** |  |  |  |
| All mixture | 1.15 (1.03, 1.28) | 1.02 (0.96, 1.08) | 0.987 |
| Female mixture | 1.08 (1.00, 1.17) | 1.00 (0.96, 1.04) | 0.974 |
| Male mixture | 1.07 (0.99, 1.16) | 1.02 (0.98, 1.06) | 0.953 |
| Coupe depression mixture | 1.11 (1.01, 1.22) | 1.00 (0.95, 1.05) | 0.915 |
| Coupe anxiety mixture | 1.12 (1.02, 1.22) | 0.99 (0.95, 1.04) | 0.935 |
| Coupe stress mixture | 1.07 (0.98, 1.18) | 1.03 (0.98, 1.08) | 0.928 |

All mixture represents the 6 components in the mixture (female/male stress, anxiety, and depression); Female mixture represents the 3 components in the mixture (female stress, anxiety, and depression); Male mixture represents the 3 components in the mixture (male stress, anxiety, and depression); Depression mixture represents the 2 components in the mixture (female/male depression); Anxiety mixture represents the 2 components in the mixture (female/male anxiety); Stress mixture represents the 2 components in the mixture (female/male stress). Data were adjusted for female age, difference between female and male ages, BMI (each partner), family income, smoking (each partner), drinking (each partner), age of menarche, pregnancy history, parity, live birth history, spontaneous abortion history, and induced abortion history.

**Supplementary Table S2. Financial subgroup analyses of the association between couples’ mental health mixture and infertility (q-gcomp model).**

| **Subgroup** | **< CNY 150 000/year** | **≥ CNY 150 000/year** | ***P* for interaction** |
| --- | --- | --- | --- |
| All mixture | 1.09 (0.97, 1.21) | 1.03 (0.97, 1.09) | 0.413 |
| Female mixture | 1.08 (0.99, 1.18) | 1.02 (0.97, 1.07) | 0.086 |
| Male mixture | 1.03 (0.94, 1.11) | 1.03 (0.99, 1.07) | 0.971 |
| Coupe depression mixture | 1.02 (0.94, 1.12) | 1.02 (0.97, 1.07) | 0.821 |
| Coupe anxiety mixture | 1.05 (0.96, 1.15) | 1.01 (0.97, 1.06) | 0.090 |
| Coupe stress mixture | 1.10 (1.01, 1.21) | 1.03 (0.98, 1.08) | 0.527 |

CNY, Chinese Yuan. All mixture represents the 6 components in the mixture (female/male stress, anxiety, and depression); Female mixture represents the 3 components in the mixture (female stress, anxiety, and depression); Male mixture represents the 3 components in the mixture (male stress, anxiety, and depression); Depression mixture represents the 2 components in the mixture (female/male depression); Anxiety mixture represents the 2 components in the mixture (female/male anxiety); Stress mixture represents the 2 components in the mixture (female/male stress). Data were adjusted for female age, difference between female and male ages, BMI (each partner), education level (each partner), smoking (each partner), drinking (each partner), age of menarche, pregnancy history, parity, live birth history, spontaneous abortion history, and induced abortion history.

**Supplementary Table S3. Couples’ educational subgroup analyses of the association between couples’ mental health mixture and infertility (q-gcomp model).**

| **Subgroup** | **Both < College** | **One of the couples < College** | **Both ≥ College** |
| --- | --- | --- | --- |
| All mixture | 1.10 (0.90, 1.35) | 1.14 (1.03, 1.27) | 1.02 (0.97, 1.08) |
| Female mixture | 1.09 (0.96, 1.24) | 1.04 (0.95, 1.12) | 1.01 (0.96, 1.05) |
| Male mixture | 0.99 (0.87, 1.12) | 1.12 (1.04, 1.22) | 1.02 (0.98, 1.06) |
| Coupe depression mixture | 1.06 (0.92, 1.23) | 1.09 (1.00, 1.20) | 1.01 (0.96, 1.05) |
| Coupe anxiety mixture | 1.07 (0.92, 1.25) | 1.11 (1.02, 1.22) | 1.01 (0.96, 1.06) |
| Coupe stress mixture | 1.03 (0.88, 1.21) | 1.07 (0.98, 1.17) | 1.03 (0.98, 1.08) |

All mixture represents the 6 components in the mixture (female/male stress, anxiety, and depression); Female mixture represents the 3 components in the mixture (female stress, anxiety, and depression); Male mixture represents the 3 components in the mixture (male stress, anxiety, and depression); Depression mixture represents the 2 components in the mixture (female/male depression); Anxiety mixture represents the 2 components in the mixture (female/male anxiety); Stress mixture represents the 2 components in the mixture (female/male stress). Data were adjusted for female age, difference between female and male ages, BMI (each partner), family income, smoking (each partner), drinking (each partner), age of menarche, pregnancy history, parity, live birth history, spontaneous abortion history, and induced abortion history.

**Supplementary Table S4. Latent profile analysis model summary table.**

| **Classes** | **LogLik** | **AIC** | **BIC** | **Entropy** | **BLRT *P* value** | **Proportion** |
| --- | --- | --- | --- | --- | --- | --- |
| 1 | -8221.166 | 16466.332 | 16524.810 | 1 | - | 1 |
| 2 | -7831.853 | 15701.706 | 15794.297 | 0.744 | 0.010 | 0.423/0.577 |
| 3 | -7598.855 | 15249.711 | 15376.413 | 0.745 | 0.010 | 0.316/0.227/0.458 |
| 4 | -7478.136 | 15022.272 | 15183.086 | 0.763 | 0.010 | 0.29/0.251/0.107/0.353 |
| 5 | -7418.865 | 14917.731 | 15112.658 | 0.741 | 0.010 | 0.17/0.151/0.107/0.318/0.255 |
| 6 | -7335.877 | 14765.754 | 14994.793 | 0.759 | 0.010 | 0.17/0.156/0.105/0.285/0.238/0.047 |
| **7** | **-7284.799** | **14677.599** | **14940.750** | **0.764** | **0.010** | **0.133/0.161/0.115/0.263/0.22/0.072/0.035** |
| 8 | -7239.288 | 14600.576 | 14897.839 | 0.760 | 0.010 | 0.137/0.129/0.13/0.189/0.223/0.052/0.112/0.028 |

LogLik, log-likelihood; AIC, Akaike information criterion; BIC, Bayesian information criterion; BLRT, bootstrap likelihood ratio test. The best-fitting model is highlighted in bold characters.

**Supplementary Table S5. Association of distinct couples’ mental health scores with their fecundability.**

|  | **n** | **FOR/RR (95% CI)** | ***P*** |
| --- | --- | --- | --- |
| **Fecundability** |  |  |  |
| Neither partner with symptoms (Group 4) | 254 | Reference | - |
| F high / M medium (Group 7) | 34 | 0.44 (0.23, 0.84) | 0.013 |
| F medium / M medium (Group 3&5) | 324 | 0.77 (0.62, 0.97) | 0.027 |
| F low / M medium (Group 1) | 128 | 0.87 (0.66, 1.15) | 0.354 |
| F medium / M low (Group 2) | 156 | 0.98 (0.75, 1.28) | 0.895 |
| F low / M high (Group 6) | 70 | 0.81 (0.56, 1.18) | 0.289 |
| **Infertility** |  |  |  |
| Neither partner with symptoms (Group 4) | 205 | Reference | - |
| F high / M medium (Group 7) | 20 | 1.17 (0.93, 1.47) | 0.162 |
| F medium / M medium (Group 3&5) | 252 | 1.07 (0.98, 1.17) | 0.112 |
| F low / M medium (Group 1) | 115 | 1.08 (0.97, 1.20) | 0.155 |
| F medium / M low (Group 2) | 122 | 1.00 (0.90, 1.12) | 0.889 |
| F low / M high (Group 6) | 54 | 1.06 (0.92, 1.23) | 0.384 |

F, female; M, male; FOR, fertility odds ratio; RR, relative risk; CI, confidence interval. Data were adjusted for female age, difference between female and male ages, BMI (each partner), family income, education level (each partner), smoking (each partner), drinking (each partner), age of menarche, pregnancy history, parity, live birth history, spontaneous abortion history, and induced abortion history.

**Supplementary Table S6. Association of distinct couples’ mental health scores with their fertility** **in different females’ educational subgroups.**

|  | **< College**  **[FOR/OR (95% CI)]** | **≥ College**  **[FOR/OR (95% CI)]** |
| --- | --- | --- |
| **Fecundability** |  |  |
| Neither partner with symptoms (Group 4) | Reference | Reference |
| F high / M medium (Group 7) | 0.39 (0.11, 1.35) | 0.51 (0.26, 1.02) |
| F medium / M medium (Group 3&5) | 0.63 (0.36, 1.10) | 0.81 (0.63, 1.03) |
| F low / M medium (Group 1) | 1.51 (0.76, 3.02) | 0.78 (0.57, 1.06) |
| F medium / M low (Group 2) | 1.19 (0.65, 2.18) | 0.91 (0.67, 1.21) |
| F low / M high (Group 6) | 0.70 (0.31, 1.56) | 0.76 (0.50, 1.16) |
| **Infertility** |  |  |
| Neither partner with symptoms (Group 4) | Reference | Reference |
| F high / M medium (Group 7) | 1.32 (0.90, 1.92) | 0.99 (0.75, 1.30) |
| F medium / M medium (Group 3&5) | 1.24 (1.01, 1.52) | 1.03 (0.93, 1.14) |
| F low / M medium (Group 1) | 0.97 (0.75, 1.25) | 1.08 (0.96, 1.22) |
| F medium / M low (Group 2) | 1.02 (0.81, 1.29) | 1.00 (0.88, 1.12) |
| F low / M high (Group 6) | 1.14 (0.85, 1.53) | 1.05 (0.89, 1.24) |

F, female; M, male; FOR, fertility odds ratio; OR, odds ratio; CI, confidence interval. Data were adjusted for female age, difference between female and male ages, BMI (each partner), family income, smoking (each partner), drinking (each partner), age of menarche, pregnancy history, parity (except in nulliparous), live birth history, spontaneous abortion history, and induced abortion history.

**Supplementary Table S7. Association of distinct couples’ mental health scores with their fertility** **in different males’ educational subgroups.**

|  | **< College**  **[FOR/OR (95% CI)]** | **≥ College**  **[FOR/OR (95% CI)]** |
| --- | --- | --- |
| **Fecundability** |  |  |
| Neither partner with symptoms (Group 4) | Reference | Reference |
| F high / M medium (Group 7) | 0.40 (0.09, 1.82) | 0.48 (0.25, 0.92) |
| F medium / M medium (Group 3&5) | 0.73 (0.41, 1.29) | 0.78 (0.61, 0.99) |
| F low / M medium (Group 1) | 1.05 (0.49, 2.26) | 0.82 (0.61, 1.10) |
| F medium / M low (Group 2) | 0.87 (0.44, 1.72) | 0.96 (0.72, 1.29) |
| F low / M high (Group 6) | 0.30 (0.08, 1.05) | 0.85 (0.58, 1.25) |
| **Infertility** |  |  |
| Neither partner with symptoms (Group 4) | Reference | Reference |
| F high / M medium (Group 7) | 1.19 (0.74, 1.93) | 1.07 (0.83, 1.37) |
| F medium / M medium (Group 3&5) | 1.03 (0.82, 1.30) | 1.07 (0.97, 1.18) |
| F low / M medium (Group 1) | 0.95 (0.70, 1.29) | 1.10 (0.98, 1.24) |
| F medium / M low (Group 2) | 1.04 (0.80, 1.35) | 0.98 (0.87, 1.10) |
| F low / M high (Group 6) | 1.23 (0.82, 1.84) | 1.04 (0.90, 1.22) |

F, female; M, male; FOR, fertility odds ratio; OR, odds ratio; CI, confidence interval. Data were adjusted for female age, difference between female and male ages, BMI (each partner), family income, smoking (each partner), drinking (each partner), age of menarche, pregnancy history, parity (except in nulliparous), live birth history, spontaneous abortion history, and induced abortion history.

**Supplementary Table S8. Association of distinct couples’ mental health scores with their fertility** **in different financial subgroups.**

|  | **< CNY 150 000/year**  **[FOR/OR (95% CI)]** | **≥ CNY 150 000/year**  **[FOR/OR (95% CI)]** |
| --- | --- | --- |
| **Fecundability** |  |  |
| Neither partner with symptoms (Group 4) | Reference | Reference |
| F high / M medium (Group 7) | 0.36 (0.10, 1.21) | 0.53 (0.27, 1.05) |
| F medium / M medium (Group 3&5) | 0.67 (0.42, 1.08) | 0.85 (0.66, 1.08) |
| F low / M medium (Group 1) | 0.72 (0.39, 1.35) | 0.90 (0.66, 1.23) |
| F medium / M low (Group 2) | 1.17 (0.62, 2.19) | 0.94 (0.70, 1.27) |
| F low / M high (Group 6) | 0.53 (0.25, 1.09) | 0.87 (0.56, 1.33) |
| **Infertility** |  |  |
| Neither partner with symptoms (Group 4) | Reference | Reference |
| F high / M medium (Group 7) | 1.52 (0.96, 2.40) | 1.01 (0.78, 1.30) |
| F medium / M medium (Group 3&5) | 1.17 (0.97, 1.41) | 1.03 (0.93, 1.14) |
| F low / M medium (Group 1) | 1.20 (0.94, 1.53) | 1.05 (0.93, 1.18) |
| F medium / M low (Group 2) | 1.13 (0.87, 1.46) | 0.98 (0.87, 1.10) |
| F low / M high (Group 6) | 1.18 (0.89, 1.55) | 1.05 (0.89, 1.24) |

CNY, Chinese Yuan; F, female; M, male; FOR, fertility odds ratio; OR, odds ratio; CI, confidence interval. Data were adjusted for female age, difference between female and male ages, BMI (each partner), education level (each partner), smoking (each partner), drinking (each partner), age of menarche, pregnancy history, parity (except in nulliparous), live birth history, spontaneous abortion history, and induced abortion history.

**Supplementary Table S9. Association of distinct couples’ mental health scores with their fertility** **in different couples’ educational subgroups.**

|  | **Both < College**  **[FOR/OR (95% CI)]** | **One of the couples < College**  **[FOR/OR (95% CI)]** | **Both ≥ College**  **[FOR/OR (95% CI)]** |
| --- | --- | --- | --- |
| **Fecundability** |  |  |  |
| Neither partner with symptoms (Group 4) | Reference | Reference | Reference |
| F high / M medium (Group 7) | 0.21 (0.02, 1.87) | 0.69 (0.20, 2.40) | 0.47 (0.22, 0.97) |
| F medium / M medium (Group 3&5) | 0.51 (0.21, 1.24) | 0.85 (0.50, 1.45) | 0.79 (0.61, 1.02) |
| F low / M medium (Group 1) | 2.10 (0.69, 6.34) | 1.02 (0.50, 2.08) | 0.78 (0.56, 1.07) |
| F medium / M low (Group 2) | 1.02 (0.39, 2.66) | 1.34 (0.69, 2.61) | 0.90 (0.66, 1.23) |
| F low / M high (Group 6) | 0.27 (0.06, 1.19) | 0.44 (0.17, 1.18) | 0.86 (0.57, 1.32) |
| **Infertility** |  |  |  |
| Neither partner with symptoms (Group 4) | Reference | Reference | Reference |
| F high / M medium (Group 7) | 1.33 (0.71, 2.47) | 1.08 (0.68, 1.71) | 1.02 (0.76, 1.37) |
| F medium / M medium (Group 3&5) | 1.10 (0.77, 1.56) | 1.17 (0.95, 1.44) | 1.03 (0.93, 1.15) |
| F low / M medium (Group 1) | 0.90 (0.58, 1.40) | 0.93 (0.70, 1.24) | 1.10 (0.97, 1.24) |
| F medium / M low (Group 2) | 1.02 (0.68, 1.51) | 0.99 (0.77, 1.28) | 0.98 (0.86, 1.12) |
| F low / M high (Group 6) | 1.04 (0.61, 1.76) | 1.38 (0.99, 1.92) | 1.01 (0.86, 1.20) |

F, female; M, male; FOR, fertility odds ratio; OR, odds ratio; CI, confidence interval. Data were adjusted for female age, difference between female and male ages, BMI (each partner), family income, smoking (each partner), drinking (each partner), age of menarche, pregnancy history, parity (except in nulliparous), live birth history, spontaneous abortion history, and induced abortion history.

**Supplementary Table S10. Multiple-exposure coefficient for the associations between couples’ mental health subdomain exposures to fecundability in elastic net regression (α = 0.1, λ = 0.389).**

| **Variables** | **ENR-β** | **Couple** | **Scale** |
| --- | --- | --- | --- |
| Did not feel as good as others | 0.012 | Female | CES-D |
| Everything was an effort | -0.073 | Female | CES-D |
| Unable to control important things | -0.067 | Female | PSS |
| Overwhelmed by difficulties | -0.169 | Female | PSS |
| Fear | -0.100 | Female | SAS |
| Panic | -0.002 | Female | SAS |
| Tremors | -0.249 | Female | SAS |
| Nightmares | -0.015 | Female | SAS |
| Trouble keeping my mind on tasks | -0.089 | Male | CES-D |
| Did not fell hopeful | 0.018 | Male | CES-D |
| Not on top of things | -0.069 | Male | PSS |
| Tremors | 0.015 | Male | SAS |
| Easy fatiguability and weakness | -0.022 | Male | SAS |
| Nausea and vomiting | -0.022 | Male | SAS |

**Supplementary Table S11. Attributable fraction of infertility caused by couples’ mental health.**

|  | **Attributable fraction (%)** | ***P*** |
| --- | --- | --- |
| Composite score (cutoff = 7) | 7.0% (-0.2%, 14.1%) | 0.055 |
| Depression-F | -3.3% (-9.3%, 2.7%) | 0.282 |
| Anxiety-F | -0.5% (-2.5%, 1.6%) | 0.657 |
| Stress-F | 7.1% (-3.3%, 17.6%) | 0.183 |
| Depression-M | 0.2% (-6.0%, 6.4%) | 0.949 |
| Anxiety-M | -1.4% (-3.0%, 0.3%) | 0.103 |
| Stress-M | 1.7% (-8.7%, 12.2%) | 0.746 |

F, female; M, male. Data were adjusted for female age, difference between female and male ages, BMI (each partner), family income, education level (each partner), smoking (each partner), drinking (each partner), age of menarche, pregnancy history, parity, live birth history, spontaneous abortion history, and induced abortion history.

**Supplementary Table S12. Educational subgroup analyses of time-dependent attributable fraction of infertility in the couples’ mental health composite score.**

|  | **< College**  **[Attributable fraction (%)]** | **≥ College**  **[Attributable fraction (%)]** |
| --- | --- | --- |
| **Female** |  |  |
| Composite score (cutoff = 7) | 10.9% (-1.6%, 23.4%) | 6.1% (-0.6%, 12.8%) |
| Depression-F | 4.7% (-10.0%, 19.3%) | -5.3% (-11.3%, 0.7%) |
| Anxiety-F | 7.3% (-7.0%, 21.6%) | -2.5% (-5.4%, 0.3%) |
| Stress-F | 14.5% (0.6%, 28.4%) | 4.9% (-4.7%, 14.6%) |
| Depression-M | 10.1% (-2.7%, 22.9%) | -2.7% (-9.1%, 3.8%) |
| Anxiety-M | 8.7% (-4.1%, 21.5%) | -4.3% (-7.6%, -1.0%) |
| Stress-M | 11.6% (-1.4%, 24.6%) | -1.1% (-11.0%, 8.7%) |
| **Male** |  |  |
| Composite score (cutoff = 7) | 10.7% (-3.2%, 24.6%) | 6.2% (-0.6%, 12.9%) |
| Depression-F | 1.5% (-14.0%, 16.9%) | -4.3% (-10.0%, 1.4%) |
| Anxiety-F | 4.0% (-10.5%, 18.6%) | -1.4% (-4.0%, 1.1%) |
| Stress-F | 11.6% (-2.6%, 25.7%) | 6.1% (-3.7%, 16.0%) |
| Depression-M | 4.8% (-13.4%, 22.9%) | -0.9% (-7.1%, 5.4%) |
| Anxiety-M | 2.7% (-16.0%, 21.4%) | -2.3% (-5.2%, 0.5%) |
| Stress-M | 6.8% (-11.3%, 24.9%) | 1.1% (-8.7%, 10.8%) |

F, female; M, male. Data were adjusted for female age, difference between female and male ages, BMI (each partner), family income, smoking (each partner), drinking (each partner), age of menarche, pregnancy history, parity, live birth history, spontaneous abortion history, and induced abortion history.

**Supplementary Table S13. Financial subgroup analyses of time-dependent attributable fraction of infertility in the couples’ mental health composite score.**

|  | **< CNY 150 000/year**  **[Attributable fraction (%)]** | **≥ CNY 150 000/year**  **[Attributable fraction (%)]** |
| --- | --- | --- |
| Composite score (cutoff = 7) | 2.8% (-12.6%, 18.3%) | 9.3% (3.0%, 15.6%) |
| Depression-F | -5.7% (-22.9%, 11.5%) | -2.4% (-8.2%, 3.5%) |
| Anxiety-F | -3.3% (-19.6%, 13.1%) | 0.2% (-2.8%, 3.2%) |
| Stress-F | 3.1% (-13.8%, 20.0%) | 6.2% (-3.0%, 15.4%) |
| Depression-M | -14.0% (-34.8%, 6.9%) | 4.8% (-0.8%, 10.3%) |
| Anxiety-M | -16.6% (-37.7%, 4.5%) | 2.6% (-0.1%, 5.3%) |
| Stress-M | -14.4% (-36.5%, 7.6%) | 4.3% (-4.5%, 13.2%) |

CNY, Chinese Yuan; F, female; M, male. Data were adjusted for female age, difference between female and male ages, BMI (each partner), education level (each partner), smoking (each partner), drinking (each partner), age of menarche, pregnancy history, parity, live birth history, spontaneous abortion history, and induced abortion history.

**Supplementary Table S14. Couples’ educational subgroup analyses of time-dependent attributable fraction of infertility in the couples’ mental health composite score.**

|  | **Both < College**  **[Attributable fraction (%)]** | **One of the couples < College**  **[Attributable fraction (%)]** | **Both ≥ College**  **[Attributable fraction (%)]** |
| --- | --- | --- | --- |
| Composite score (cutoff = 7) | 19.0% (1.4%, 36.6%) | 7.8% (-10.4%, 26.0%) | 5.3% (-1.2%, 11.8%) |
| Depression-F | 12.2% (-8.0%, 32.3%) | -3.3% (-23.1%, 16.5%) | -5.5% (-11.6%, 0.6%) |
| Anxiety-F | 14.7% (-4.7%, 34.1%) | -0.5% (-19.7%, 18.7%) | -2.7% (-6.2%, 0.8%) |
| Stress-F | 21.4% (3.1%, 39.7%) | 7.3% (-10.8%, 25.4%) | 4.7% (-4.6%, 14.1%) |
| Depression-M | 15.3% (-5.0%, 35.5%) | 2.6% (-17.5%, 22.8%) | -2.9% (-9.5%, 3.8%) |
| Anxiety-M | 13.4% (-7.7%, 34.4%) | 0.9% (-19.4%, 21.2%) | -4.3% (-8.6%, -0.1%) |
| Stress-M | 17.1% (-2.6%, 36.9%) | 4.6% (-15.5%, 24.8%) | -0.9% (-10.6%, 8.8%) |

F, female; M, male. Data were adjusted for female age, difference between female and male ages, BMI (each partner), family income, smoking (each partner), drinking (each partner), age of menarche, pregnancy history, parity, live birth history, spontaneous abortion history, and induced abortion history.

**Supplementary Table S15. Associations between each partner’s mental health and couple fecundability with imputed data.**

|  | **FORs (95% CI)** | **ORs (95% CI)** |
| --- | --- | --- |
| **1 SD increase** |  |  |
| Depression-F | 0.92 (0.84, 1.00) | 1.04 (0.89, 1.22) |
| Anxiety-F | 0.94 (0.86, 1.02) | 1.00 (0.86, 1.17) |
| Stress-F | 0.88 (0.81, 0.96) | 1.14 (0.97, 1.33) |
| Depression-M | 0.95 (0.88, 1.04) | 1.08 (0.92, 1.26) |
| Anxiety-M | 0.90 (0.83, 0.99) | 1.17 (1.00, 1.36) |
| Stress-M | 0.93 (0.86, 1.02) | 1.08 (0.92, 1.26) |

F, female; M, male; FOR, fertility odds ratio; OR, odds ratio; CI, confidence interval; SD, standard deviation. For females, data were adjusted for their age, BMI, family income, education level, smoking, drinking, age of menarche, pregnancy history, parity, live birth history, spontaneous abortion history, and induced abortion history. For males, data were adjusted for their age, BMI, family income, education level, smoking, drinking; their partner’s pregnancy history, parity, live birth history, spontaneous abortion history, and induced abortion history.

**Supplementary Table S16. Associations between each partner’s mental health and couple subfecundability and subfertility.**

|  | **Subfecundability**  **FORs (95% CI)** | **Subfertility**  **ORs (95% CI)** |
| --- | --- | --- |
| **1 SD increase** |  |  |
| Depression-F | 0.89 (0.81, 0.98) | 1.14 (0.96, 1.35) |
| Anxiety-F | 0.89 (0.81, 0.98) | 1.12 (0.95, 1.34) |
| Stress-F | 0.86 (0.78, 0.95) | 1.16 (0.99, 1.38) |
| Depression-M | 0.96 (0.88, 1.05) | 0.99 (0.84, 1.17) |
| Anxiety-M | 0.93 (0.85, 1.02) | 1.08 (0.91, 1.27) |
| Stress-M | 0.94 (0.86, 1.02) | 1.00 (0.85, 1.19) |

F, female; M, male; FOR, fertility odds ratio; OR, odds ratio; CI, confidence interval; SD, standard deviation. For females, data were adjusted for their age, BMI, family income, education level, smoking, drinking, age of menarche, pregnancy history, parity, live birth history, spontaneous abortion history, and induced abortion history. For males, data were adjusted for their age, BMI, family income, education level, smoking, drinking; their partner’s pregnancy history, parity, live birth history, spontaneous abortion history, and induced abortion history.

**Supplementary Table S17. Associations between each partner’s mental health and their fecundability in females with regular menstrual cycle and nulliparous couples.**

|  | **Regular menstrual cycle** | | **Nulliparous** | |
| --- | --- | --- | --- | --- |
|  | Fecundability FORs  (95% CI) | Infertility ORs  (95% CI) | Fecundability FORs  (95% CI) | Infertility ORs  (95% CI) |
| **1 SD increase** |  |  |  |  |
| Depression-F | 0.92 (0.82, 1.02) | 1.13 (0.93, 1.37) | 0.90 (0.82, 1.00) | 1.08 (0.90, 1.29) |
| Anxiety-F | 0.94 (0.85, 1.04) | 1.05 (0.86, 1.28) | 0.92 (0.84, 1.02) | 1.06 (0.88, 1.27) |
| Stress-F | 0.95 (0.86, 1.04) | 1.15 (0.95, 1.40) | 0.94 (0.86, 1.03) | 1.16 (0.98, 1.39) |
| Depression-M | 0.97 (0.88, 1.07) | 1.04 (0.87, 1.24) | 0.95 (0.87, 1.04) | 1.07 (0.91, 1.26) |
| Anxiety-M | 0.92 (0.81, 1.05) | 1.26 (0.99, 1.61) | 0.91 (0.83, 1.00) | 1.17 (1.99, 1.38) |
| Stress-M | 0.87 (0.78, 0.97) | 1.07 (0.89, 1.28) | 0.87 (0.79, 0.96) | 1.06 (0.90, 1.25) |

F, female; M, male; FOR, fertility odds ratio; OR, odds ratio; CI, confidence interval; SD, standard deviation. For females, data were adjusted for their age, BMI, family income, education level, smoking, drinking, age of menarche, pregnancy history, parity, live birth history, spontaneous abortion history, and induced abortion history. For males, data were adjusted for their age, BMI, family income, education level, smoking, drinking; their partner’s pregnancy history, parity, live birth history, spontaneous abortion history, and induced abortion history.

**Supplementary Table S18. Associations between couples’ depression, anxiety, and stress and their fecundability with data imputation.**

|  | **FORs (95% CI)** | **ORs (95% CI)** |
| --- | --- | --- |
| **All** | 0.82 (0.73, 0.94) | 1.04 (0.99, 1.10) |
| Female | 0.89 (0.81, 0.98) | 1.02 (0.98, 1.05) |
| Male | 0.91 (0.83, 0.99) | 1.03 (0.99, 1.06) |
| **Depression** | 0.88 (0.80, 0.98) | 1.02 (0.98, 1.06) |
| None | Reference | Reference |
| Male only | 0.88 (0.68, 1.12) | 1.03 (0.93, 1.13) |
| Female only | 0.92 (0.74, 1.15) | 0.97 (0.89, 1.07) |
| Both | 0.95 (0.70, 1.29) | 1.01 (0.89, 1.14) |
| **Anxiety** | 0.90 (0.81, 1.00) | 1.02 (0.98, 1.06) |
| None | Reference | Reference |
| Male only | 1.31 (0.75, 2.31) | 0.87 (0.69, 1.11) |
| Female only | 0.83 (0.56, 1.25) | 0.94 (0.80, 1.12) |
| Both | 0.45 (0.06, 3.32) | 1.33 (0.68, 2.60) |
| **Stress** | 0.86 (0.77, 0.95) | 1.04 (1.00, 1.08) |
| None | Reference | Reference |
| Male only | 0.91 (0.71, 1.16) | 1.08 (0.97, 1.19) |
| Female only | 0.86 (0.69, 1.09) | 1.07 (0.98, 1.17) |
| Both | 0.79 (0.62, 0.99) | 1.07 (0.98, 1.18) |

FOR, fertility odds ratio; OR, odds ratio; CI, confidence interval. All, Female, Male, Depression, Anxiety, and Stress represent the associations between mixtures of different couples’ mental health component scores and fertility, calculated using the q-gcomp model. All represents the 6 components in the mixture (female/male stress, anxiety, and depression); Female represents the 3 components in the mixture (female stress, anxiety, and depression); Male represents the 3 components in the mixture (male stress, anxiety, and depression); Depression represents the 2 components in the mixture (female/male depression); Anxiety represents the 2 components in the mixture (female/male anxiety); Stress represents the 2 components in the mixture (female/male stress). None, male only, female only, and both represent subgroups defined by cross-classification according to the cutoff values of the corresponding dimension, analyzed using Cox or logistic regression models with None as the reference group. Data were adjusted for female age, difference between female and male ages, BMI (each partner), family income, smoking (each partner), drinking (each partner), age of menarche, pregnancy history, parity, live birth history, spontaneous abortion history, and induced abortion history.

**Supplementary Table S19. Associations of couples’ depression, anxiety, and stress with their subfecundability and subfertility.**

|  | **Subfecundability**  **FORs (95% CI)** | **Subfertility**  **ORs (95% CI)** |
| --- | --- | --- |
| **All** | 0.83 (0.73, 0.95) | 1.04 (0.98, 1.09) |
| Female | 0.88 (0.80, 0.97) | 1.03 (0.99, 1.07) |
| Male | 0.92 (0.84, 1.02) | 1.00 (0.96, 1.04) |
| **Depression** | 0.83 (0.73, 0.95) | 1.02 (0.98, 1.07) |
| None | Reference | Reference |
| Male only | 0.97 (0.75, 1.24) | 0.96 (0.87, 1.07) |
| Female only | 0.97 (0.77, 1.21) | 1.01 (0.91, 1.11) |
| Both | 1.05 (0.77, 1.44) | 1.00 (0.87, 1.14) |
| **Anxiety** | 0.90 (0.81, 1.00) | 1.02 (0.97, 1.06) |
| None | Reference | Reference |
| Male only | 1.71 (0.97, 3.01) | 0.98 (0.75, 1.28) |
| Female only | 0.76 (0.51, 1.15) | 1.13 (0.95, 1.35) |
| Both | 0.50 (0.07, 3.68) | 1.04 (0.52, 2.10) |
| **Stress** | 0.86 (0.77, 0.95) | 1.02 (0.98, 1.07) |
| None | Reference | Reference |
| Male only | 0.96 (0.75, 1.24) | 0.97 (0.87, 1.08) |
| Female only | 0.88 (0.70, 1.11) | 1.02 (0.93, 1.13) |
| Both | 0.77 (0.60, 0.97) | 1.05 (0.95, 1.16) |

FOR, fertility odds ratio; OR, odds ratio; CI, confidence interval. All, Female, Male, Depression, Anxiety, and Stress represent the associations between mixtures of different couples’ mental health component scores and fertility, calculated using the q-gcomp model. All represents the 6 components in the mixture (female/male stress, anxiety, and depression); Female represents the 3 components in the mixture (female stress, anxiety, and depression); Male represents the 3 components in the mixture (male stress, anxiety, and depression); Depression represents the 2 components in the mixture (female/male depression); Anxiety represents the 2 components in the mixture (female/male anxiety); Stress represents the 2 components in the mixture (female/male stress). None, male only, female only, and both represent subgroups defined by cross-classification according to the cutoff values of the corresponding dimension, analyzed using Cox or logistic regression models with None as the reference group. Data were adjusted for female age, difference between female and male ages, BMI (each partner), family income, smoking (each partner), drinking (each partner), age of menarche, pregnancy history, parity, live birth history, spontaneous abortion history, and induced abortion history.

**Supplementary Table S20. Associations of couples’ depression, anxiety, and stress with their fecundability in females with regular menstrual cycle and nulliparous couples.**

|  | **Regular menstrual cycle** | | **Nulliparous** | |
| --- | --- | --- | --- | --- |
|  | Fecundability FORs  (95% CI) | Infertility ORs  (95% CI) | Fecundability FORs  (95% CI) | Infertility ORs  (95% CI) |
| **All** | 0.87 (0.64, 1.19) | 1.05 (0.92, 1.19) | 0.83 (0.72, 0.95) | 1.04 (0.99, 1.10) |
| Female | 0.97 (0.76, 1.22) | 1.02 (0.93, 1.12) | 0.89 (0.80, 0.98) | 1.01 (0.98, 1.06) |
| Male | 0.89 (0.71, 1.11) | 1.03 (0.94, 1.12) | 0.92 (0.83, 1.02) | 1.02 (0.98, 1.07) |
| **Depression** | 0.91 (0.70, 1.18) | 1.02 (0.92, 1.14) | 0.90 (0.80, 1.00) | 1.02 (0.97, 1.06) |
| None | Reference | Reference | Reference | Reference |
| Male only | 0.84 (0.63, 1.12) | 1.05 (0.94, 1.17) | 0.95 (0.74, 1.23) | 1.01 (0.91, 1.12) |
| Female only | 1.00 (0.78, 1.28) | 0.96 (0.87, 1.07) | 1.00 (0.78, 1.28) | 0.93 (0.84, 1.03) |
| Both | 1.25 (0.90, 1.76) | 0.93 (0.81, 1.07) | 1.07 (0.76, 1.49) | 0.95 (0.83, 1.09) |
| **Anxiety** | 0.93 (0.72, 1.20) | 1.01 (0.92, 1.12) | 0.91 (0.82, 1.02) | 1.01 (0.97, 1.06) |
| None | Reference | Reference |  |  |
| Male only | 1.28 (0.59, 2.75) | 0.86 (0.62, 1.20) | 1.87 (1.06, 3.30) | 0.79 (0.61, 1.02) |
| Female only | 0.88 (0.56, 1.40) | 0.96 (0.79, 1.17) | 0.80 (0.52, 1.22) | 0.95 (0.80, 1.13) |
| Both | 0.46 (0.06, 3.42) | 1.37 (0.70, 2.68) | NA | 1.95 (0.77, 4.94) |
| **Stress** | 0.89 (0.68, 1.15) | 1.06 (0.95, 1.17) | 0.85 (0.76, 0.95) | 1.04 (1.00, 1.09) |
| None | Reference | Reference | Reference | Reference |
| Male only | 0.93 (0.70, 1.23) | 1.05 (0.93, 1.17) | 0.87 (0.67, 1.14) | 1.09 (0.98, 1.21) |
| Female only | 0.86 (0.66, 1.11) | 1.06 (0.95, 1.18) | 0.83 (0.65, 1.06) | 1.07 (0.97, 1.18) |
| Both | 0.80 (0.62, 1.04) | 1.05 (0.95, 1.17) | 0.75 (0.58, 0.96) | 1.08 (0.97, 1.19) |

FOR, fertility odds ratio; OR, odds ratio; CI, confidence interval; NA, not applicable. All, Female, Male, Depression, Anxiety, and Stress represent the associations between mixtures of different couples’ mental health component scores and fertility, calculated using the q-gcomp model. All represents the 6 components in the mixture (female/male stress, anxiety, and depression); Female represents the 3 components in the mixture (female stress, anxiety, and depression); Male represents the 3 components in the mixture (male stress, anxiety, and depression); Depression represents the 2 components in the mixture (female/male depression); Anxiety represents the 2 components in the mixture (female/male anxiety); Stress represents the 2 components in the mixture (female/male stress). None, male only, female only, and both represent subgroups defined by cross-classification according to the cutoff values of the corresponding dimension, analyzed using Cox or logistic regression models with None as the reference group. Data were adjusted for female age, difference between female and male ages, BMI (each partner), family income, smoking (each partner), drinking (each partner), age of menarche, pregnancy history, parity, live birth history, spontaneous abortion history, and induced abortion history.

**Supplementary Table S21. Association of distinct couples’ mental health scores with their fertility with data imputation.**

|  | **FOR/OR (95% CI)** | ***P*** |
| --- | --- | --- |
| **Fecundability** |  |  |
| Neither partner with symptoms (Group 4) | Reference | - |
| F high / M medium (Group 7) | 0.47 (0.26, 0.86) | 0.014 |
| F medium / M medium (Group 3&5) | 0.79 (0.64, 0.99) | 0.043 |
| F low / M medium (Group 1) | 0.87 (0.66, 1.15) | 0.351 |
| F medium / M low (Group 2) | 0.95 (0.73, 1.23) | 0.712 |
| F low / M high (Group 6) | 0.75 (0.52, 1.08) | 0.127 |
| **Infertility** |  |  |
| Neither partner with symptoms (Group 4) | Reference | - |
| F high / M medium (Group 7) | 1.11 (0.89, 1.38) | 0.321 |
| F medium / M medium (Group 3&5) | 1.06 (0.97, 1.16) | 0.146 |
| F low / M medium (Group 1) | 1.07 (0.96, 1.19) | 0.188 |
| F medium / M low (Group 2) | 1.00 (0.90, 1.12) | 0.880 |
| F low / M high (Group 6) | 1.08 (0.94, 1.24) | 0.269 |

F, female; M, male; FOR, fertility odds ratio; OR, odds ratio; CI, confidence interval. Data were adjusted for female age, difference between female and male ages, BMI (each partner), family income, education level (each partner), smoking (each partner), drinking (each partner), age of menarche, pregnancy history, parity, live birth history, spontaneous abortion history, and induced abortion history.

**Supplementary Table S22. Association of distinct couples’ mental health scores with their subfecundability and subfertility.**

|  | **FOR/OR (95% CI)** | ***P*** |
| --- | --- | --- |
| **Subfecundability** |  |  |
| Neither partner with symptoms (Group 4) | Reference | - |
| F high / M medium (Group 7) | 0.42 (0.22, 0.81) | 0.009 |
| F medium / M medium (Group 3&5) | 0.78 (0.63, 0.98) | 0.038 |
| F low / M medium (Group 1) | 0.97 (0.73, 1.28) | 0.845 |
| F medium / M low (Group 2) | 0.98 (0.75, 1.29) | 0.933 |
| F low / M high (Group 6) | 0.83 (0.57, 1.20) | 0.336 |
| **Subfertility** |  |  |
| Neither partner with symptoms (Group 4) | Reference | - |
| F high / M medium (Group 7) | 1.20 (0.94, 1.53) | 0.126 |
| F medium / M medium (Group 3&5) | 1.06 (0.96, 1.17) | 0.190 |
| F low / M medium (Group 1) | 1.01 (0.90, 1.14) | 0.763 |
| F medium / M low (Group 2) | 0.98 (0.87, 1.10) | 0.763 |
| F low / M high (Group 6) | 0.97 (0.83, 1.13) | 0.735 |

F, female; M, male; FOR, fertility odds ratio; OR, odds ratio; CI, confidence interval. Data were adjusted for female age, difference between female and male ages, BMI (each partner), family income, education level (each partner), smoking (each partner), drinking (each partner), age of menarche, pregnancy history, parity, live birth history, spontaneous abortion history, and induced abortion history.

**Supplementary Table S23. Association of distinct couples’ mental health scores with their fertility in females with regular menstrual cycle and nulliparous couples.**

|  | **Regular menstrual cycle**  **[FOR/OR (95% CI)]** | **Nulliparous**  **[FOR/OR (95% CI)]** |
| --- | --- | --- |
| **Fecundability** |  |  |
| Neither partner with symptoms (Group 4) | Reference | Reference |
| F high / M medium (Group 7) | 0.44 (0.21, 0.90) | 0.38 (0.19, 0.76) |
| F medium / M medium (Group 3&5) | 0.83 (0.65, 1.07) | 0.77 (0.60, 0.97) |
| F low / M medium (Group 1) | 0.96 (0.70, 1.30) | 0.86 (0.64, 1.16) |
| F medium / M low (Group 2) | 1.02 (0.76, 1.36) | 0.97 (0.73, 1.30) |
| F low / M high (Group 6) | 0.68 (0.44, 1.06) | 0.78 (0.53, 1.16) |
| **Infertility** |  |  |
| Neither partner with symptoms (Group 4) | Reference | Reference |
| F high / M medium (Group 7) | 1.19 (0.92, 1.55) | 1.20 (0.94, 1.52) |
| F medium / M medium (Group 3&5) | 1.05 (0.95, 1.16) | 1.07 (0.98, 1.18) |
| F low / M medium (Group 1) | 1.06 (0.94, 1.20) | 1.08 (0.96, 1.21) |
| F medium / M low (Group 2) | 1.00 (0.89, 1.13) | 0.99 (0.88, 1.11) |
| F low / M high (Group 6) | 1.12 (0.95, 1.32) | 1.08 (0.93, 1.26) |

F, female; M, male; FOR, fertility odds ratio; OR, odds ratio; CI, confidence interval. Data were adjusted for female age, difference between female and male ages, BMI (each partner), family income, education level (each partner), smoking (each partner), drinking (each partner), age of menarche, pregnancy history, parity (except in nulliparous), live birth history, spontaneous abortion history, and induced abortion history.

**Supplementary Table S24. Attributable fraction of infertility caused by couples’ mental health with data imputation.**

|  | **Attributable fraction (%)** | ***P*** |
| --- | --- | --- |
| Composite score (cutoff = 7) | 8.0% (1.1%, 14.8%) | 0.023 |
| Depression-F | -3.0% (-8.8%, 2.9%) | 0.317 |
| Anxiety-F | -0.5% (-2.5%, 1.5%) | 0.625 |
| Stress-F | 5.4% (-4.8%, 15.7%) | 0.299 |
| Depression-M | 1.2% (-4.3%, 6.7%) | 0.670 |
| Anxiety-M | -0.6% (-2.2%, 0.9%) | 0.438 |
| Stress-M | 4.9% (-4.5%, 14.3%) | 0.306 |

F, female; M, male. Data were adjusted for female age, difference between female and male ages, BMI (each partner), family income, education level (each partner), smoking (each partner), drinking (each partner), age of menarche, pregnancy history, parity, live birth history, spontaneous abortion history, and induced abortion history.

**Supplementary Table S25. Attributable fraction of subfertility caused by couples’ mental health.**

|  | **Attributable fraction (%)** | ***P*** |
| --- | --- | --- |
| Composite score (cutoff = 7) | 3.8% (-0.6%, 8.3%) | 0.093 |
| Depression-F | 0.7% (-3.1%, 4.6%) | 0.712 |
| Anxiety-F | 0.9% (-0.4%, 2.2%) | 0.173 |
| Stress-F | 4.9% (-1.9%, 11.6%) | 0.156 |
| Depression-M | -1.3% (-5.3%, 2.7%) | 0.510 |
| Anxiety-M | -0.4% (-1.6%, 0.8%) | 0.497 |
| Stress-M | -1.5% (-8.2%, 5.2%) | 0.663 |

F, female; M, male. Data were adjusted for female age, difference between female and male ages, BMI (each partner), family income, education level (each partner), smoking (each partner), drinking (each partner), age of menarche, pregnancy history, parity, live birth history, spontaneous abortion history, and induced abortion history.

**Supplementary Table S26. Attributable fraction of infertility caused by couples’ mental health in females with regular menstrual cycle and nulliparous couples.**

|  | **Regular menstrual cycle**  **[Attributable fraction (%)]** | **Nulliparous**  **[Attributable fraction (%)]** |
| --- | --- | --- |
| Composite score (cutoff = 7) | 7.1% (0.3%, 13.9%) | 7.0% (0.1%, 13.9%) |
| Depression-F | -3.1% (-9.3%, 3.1%) | -3.1% (-8.6%, 2.3%) |
| Anxiety-F | -0.2% (-3.2%, 2.8%) | -0.5% (-2.6%, 1.6%) |
| Stress-F | 7.4% (-2.2%, 16.9%) | 7.1% (-3.0%, 17.1%) |
| Depression-M | 0.9% (-5.2%, 7.0%) | 0.2% (-6.1%, 6.5%) |
| Anxiety-M | -0.7% (-3.6%, 2.3%) | -1.4% (-3.2%, 0.3%) |
| Stress-M | 2.4% (-7.2%, 12.1%) | 1.7% (-8.5%, 12.0%) |

F, female; M, male. Data were adjusted for female age, difference between female and male ages, BMI (each partner), family income, education level (each partner), smoking (each partner), drinking (each partner), age of menarche, pregnancy history, parity (except in nulliparous), live birth history, spontaneous abortion history, and induced abortion history.
